# Structure, Function, and Phylogeny of the Mating Locus in the *Rhizopus oryzae* Complex

**DOI:** 10.1371/journal.pone.0015273

**Published:** 2010-12-09

**Authors:** Andrii P. Gryganskyi, Soo Chan Lee, Anastasia P. Litvintseva, Matthew E. Smith, Gregory Bonito, Teresita M. Porter, Iryna M. Anishchenko, Joseph Heitman, Rytas Vilgalys

**Affiliations:** 1 Department of Biology, Duke University, Durham, North Carolina, United States of America; 2 Department of Mycology, M.G. Kholodny Institute of Botany, National Academy of Sciences of Ukraine, Kyiv, Ukraine; 3 Department of Molecular Genetics and Microbiology, Duke University Medical Center, Durham, North Carolina, United States of America; Institute of Infectious Disease and Molecular Medicine, South Africa

## Abstract

The *Rhizopus oryzae* species complex is a group of zygomycete fungi that are common, cosmopolitan saprotrophs. Some strains are used beneficially for production of Asian fermented foods but they can also act as opportunistic human pathogens. Although *R. oryzae* reportedly has a heterothallic (+/−) mating system, most strains have not been observed to undergo sexual reproduction and the genetic structure of its mating locus has not been characterized. Here we report on the mating behavior and genetic structure of the mating locus for 54 isolates of the *R. oryzae* complex. All 54 strains have a mating locus similar in overall organization to *Phycomyces blakesleeanus* and *Mucor circinelloides* (Mucoromycotina, Zygomycota). In all of these fungi, the minus (−) allele features the *SexM* high mobility group (HMG) gene flanked by an RNA helicase gene and a TP transporter gene (TPT). Within the *R. oryzae* complex, the plus (+) mating allele includes an inserted region that codes for a *BTB/POZ* domain gene and the *SexP* HMG gene. Phylogenetic analyses of multiple genes, including the mating loci (HMG, TPT, RNA helicase), ITS1-5.8S-ITS2 rDNA, *RPB2*, and *LDH* genes, identified two distinct groups of strains. These correspond to previously described sibling species *R. oryzae sensu stricto* and *R. delemar*. Within each species, discordant gene phylogenies among multiple loci suggest an outcrossing population structure. The hypothesis of random-mating is also supported by a 50∶50 ratio of plus and minus mating types in both cryptic species. When crossed with tester strains of the opposite mating type, most isolates of *R. delemar* failed to produce zygospores, while isolates of *R. oryzae* produced sterile zygospores. In spite of the reluctance of most strains to mate *in vitro*, the conserved *sex* locus structure and evidence for outcrossing suggest that a normal sexual cycle occurs in both species.

## Introduction

*Rhizopus oryzae* is a complex of closely related, heterothallic species [1], [2], [3], [4] that are common, cosmopolitan saprotrophs in soil, dung, and rotting vegetation [5], [6], [7], [8]. Strains of the *R. oryzae* complex have been used for centuries as fermented food starters for the production of tempeh and other Asian foods [9], [10], [11], [12], [13], [14] but species in this group can also act as opportunistic, invasive animal and human pathogens that cause deadly infections in immuno-compromised individuals [15], [16], [17], [18], [19], [20].

Like other fungi belonging to subphylum Mucoromycotina, members of the *R. oryzae* complex have a bipolar mating system [21], [22]. Compatible (+) and (−) mating types are recognizable by their mating behavior and subsequent production of meiospores (zygospores). However, mating behavior is not always consistent among different strains. Furthermore, zygospore germination and progeny development within the genus *Rhizopus* appears to be rare and has only been documented in a few cases [19], [21].

The mating behavior of *Rhizopus* species represents a distinct pattern of zygospore production among Mucoralean fungi known as the “*Rhizopus* pattern”. In this type of zygospore formation, some nuclei fuse while others degenerate and meiosis is delayed until zygospore germination [21], [23]. Zygospores have been observed in both heterothallic and homothallic *Rhizopus* species [3], [4] but in most cases they do not germinate. Although zygospore germination is rare, it has been documented for *R. stolonifer* [24], [25], [26]. In addition, some *Rhizopus* species produce azygospores, which are thought to be asexual. They are morphologically similar to zygospores but develop in the absence of a mating partner.

Recent studies of sexual reproduction in the zygomycete *Phycomyces blakesleeanus* revealed that the mating system of this species is regulated by divergent alleles of a single gene: *SexM* (−) and *SexP* (+) [16]. The *sex* gene is a member of the high mobility genes (HMG) family and is located between two flanking genes that code for a triose-phosphate transporter homolog (TPT) and an RNA helicase [16]. A similar *sex* locus structure was also reported in another zygomycete, *Mucor circinelloides* [27] and was predicted for *R. oryzae* based on BLAST analysis of the publicly available genome of strain RA99-880 [16]. A recent study also found a similar *sex*-related locus in three Microsporidian species, which are believed to be closely related to the zygomycetes [27], [28].

In this study, we characterized the *sex* locus in six strains of the *R. oryzae* species complex (four (−) strains and two (+) strains) and examined the genetic structure of the *sex* locus in a larger collection of clinical, industrial, and environmental isolates. We identified the most conserved regions in the *sex* locus and designed primers for three key regions: the high mobility group (HMG) gene regions, the triose-phosphate transporter (TPT) region, and the RNA helicase region. We used these primers to amplify fragments of the *sex* locus from 40 additional isolates and used these DNA sequences to study phylogenetic relationships among loci. Here we compare the phylogenetic pattern from the *sex* genes with those from several other commonly sequenced DNA loci (ITS1-5.8S-ITS2 and 28S rDNA, *RPB2*, mtSSU) and demonstrate that the *sex* loci are informative for differentiating the cryptic sibling species *R. oryzae s. s.* and *R. delemar*. We also compare the *sex* locus structure of *R. oryzae* (+) and (−) strains with those of the two mucoralean fungi *Phycomyces blakesleeanus* and *Mucor circinelloides* and show that the *sex* locus of all three taxa has a similar overall arrangement.

## Results

### Mating tests produce sexual spores but not progeny

We repeated the mating tests reported by Schipper [3] using the same strains: (+) CBS346.36 and (−) CBS110.17, CBS112.07 (holotype culture), CBS127.08, CBS148.22, CBS257.28, CBS264.28, CBS266.30, CBS329.47, and CBS382.52. We were not able to include the holotype culture of *Rhizopus delemar* (CBS120.12, GenBank # AB181318), but we have included the *R. delemar* strain NRRL21447, which has an ITS1-5.8S-ITS2 sequence that is identical to that of the type culture (Fig. S1A). As observed by Schipper, zygospores formed in all mating reactions except those performed with (−) strain CBS257.28.

We also tested additional *R. oryzae* isolates from the NRRL and Duke collections using the tester strains CBS346.36 (+) and CBS112.07 (−). These mating tests identified six additional compatible isolates that were capable of producing zygospores when paired with tester strains of the opposite mating type (Table 1, Table S3). Self-pairings or pairings with senescent cultures consistently failed to produce zygospores. Mating test results were not influenced by medium type, medium nutrient concentration, or Petri plate size. Most strains did not produce zygospores with either “plus” or “minus” testers under the conditions we tested. The genome strain RA99-880 also failed to produce zygospores in all mating tests with any of the 55 strains used in this study.

**Table 1:** Isolates of the *Rhizopus oryzae* complex used in this study.

Collection #	Zygospores	Mating type	Origin and notes
***R. oryzae s. s.***			
CBS112.07^3, 4, 5^	yes	minus 1, 2	human pathogen
CBS127.08^3^	yes	minus 1, 2	human pathogen
CBS148.22	yes	minus 1, 2	as *R. tonkinensis*, lactic acid
CBS257.28^5^	yes	minus 1, 2	as *R. formosaensis*, fermented food
CBS266.30	yes	minus 1, 2	as *R. fusiformis*, on *Brassica* root
CBS346.36^3, 5^	yes	plus 1, 2	non pathogenic
CBS382.52	yes	minus 1, 2	produces steroids
Duke166.02	no	plus 2	human pathogen
Duke99-133	no	n. d.	human pathogen
Duke99-892	no	plus 2	human pathogen
NRRL395	no	plus 2	tempeh
NRRL1891	no	plus 2	Hildebrandt
NRRL1897	yes	minus 1, 2	grain
NRRL2908	yes	plus 1, 2	Chinese yeasts
NRRL3142	no	minus 2	Chinese yeasts
NRRL5833	no	plus 2	produces 6-azauridine
NRRL5834	no	minus 2	barley
NRRL6142	no	minus 2	parsnip, produces steroids
NRRL6311	no	minus 2	soft red wheat
NRRL10206	no	plus 2	human pathogen
NRRL21251	no	plus 2	human pathogen
NRRL21789	yes	plus 2	human pathogen
NRRL28631	no	plus 2	human pathogen
NRRLA-336	yes	minus 1, 2	wine cake
NRRLA-10884	no	minus 1	human pathogen
NRRLA-13142	no	minus 2	human pathogen
NRRLA-13440	yes	minus 1, 2	human pathogen
***R. delemar***			
CBS329.47	yes	minus 1, 2	tempeh, produces pectinase
NRRL1528	no	minus 2	
NRRL1548	yes	minus 1, 2	
NRRL1549	no	plus 2	
NRRL1551^3^	no	minus 2	
NRRL1552	no	minus 2	
NRRL2005	no	plus 2	produces fumaric acid
NRRL3562	no	plus 2	tempeh
NRRL3563	no	plus 2	tempeh
NRRL13098	no	minus 2	tapioca (tapai-ubi)
NRRL21447	no	minus 2	human pathogen
NRRLA-16456	no	minus 2	human pathogen
RA99-880^3^	no	plus 2	human pathogen

1 determined in mating assays.

2 determined by sequencing the *sex* locus.

3 Strains for which the sex locus and flanking genes have been sequenced.

4 Type culture.

5 Tester strains used in mating assays.

n. d. – not determined.

Where mating occurred, zygospores developed within two to three weeks. Although complete darkness is required for successful mating tests of some zygomycetes (e. g. *Mucor* spp.) [29], we determined that *R. oryzae* group isolates formed zygospores in partial light or in complete darkness. During mating, most zygomycetes form a straight line of zygospores at the interface between the two compatible strains, which has been reported previously for *R. oryzae* [30] (C. Skory, personal communication). However, we did not observe the typical straight line of zygospores and instead found them diffusely distributed across the plate within ca 4 to 5 cm of the inoculation points. The majority of the zygospores were observed on the side of the Petri plate where the (+) strain was growing.

**Figure 1:**
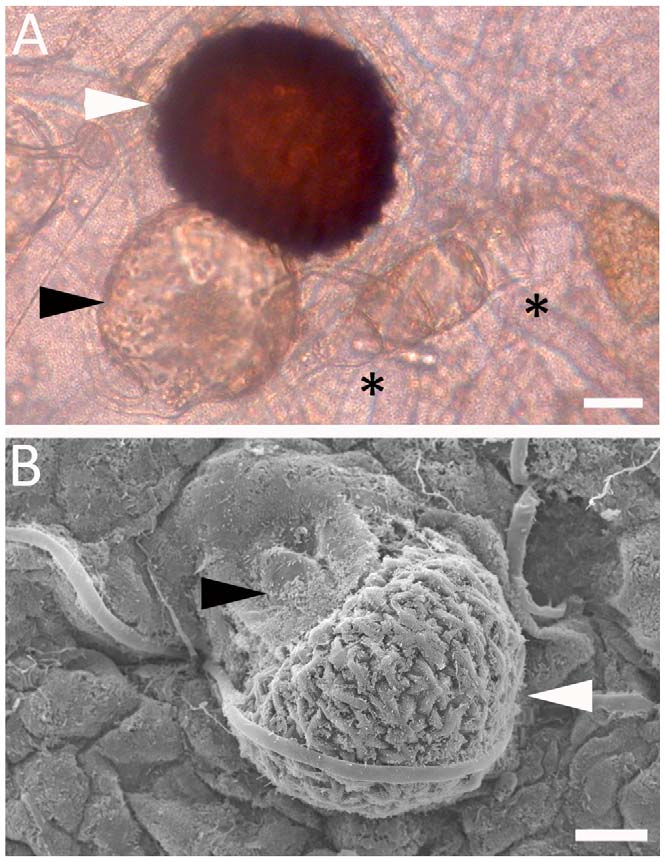
Zygospores of *Rhizopus oryzae*. A cross between CBS346.36 (+) and CBS110.17 (−) was analyzed with light microscopy (A) and scanning electron microscopy (B). Zygospores (white arrow heads) are attached to an asymmetric suspensor (black arrow heads). Scale = 20 µm. Asterisks indicate conjugated hyphae during early stage of zygospore formation.

Under the light microscope, initiation of individual zygospores between two conjugating hyphae could be observed. Conjugating hyphae were consistently shorter and thicker than vegetative hyphae, and were separated from vegetative hyphae by visible septae. After zygospore formation, conjugating hyphae developed into asymmetric suspensor cells (Fig. 1) [3]. Zygospores were morphologically variable and ranged from 60–140 µm in diameter. Zygospore shape ranged from round to flat with stellate conical projections (Fig. S2A) and color ranged from reddish brown to dark brown. As in previous mating studies of *R. oryzae s. l.*, we also observed large, central vacuoles inside many zygospores [21].

Contrary to published results for *R. stolonifer* [25], [26], we were unable to stimulate germination of zygospores into germosporangia under any of the experimental conditions tested. Gentle crushing of exospore (outer zygospore wall) [31] resulted in the release of protoplast material, which developed into a vegetative mycelium (Fig. S2C). Fourteen single spore isolates were germinated and grown in 100 ml of PD medium overnight. DNA was extracted from these cultures as previously described for standard isolates. Using the described primers, we obtained DNA sequences for the *sex* locus of these isolates and determined that all 14 single spore isolates had only the (−) mating type.

### Structure of the mating locus in *R. oryzae*

**Figure 2:**
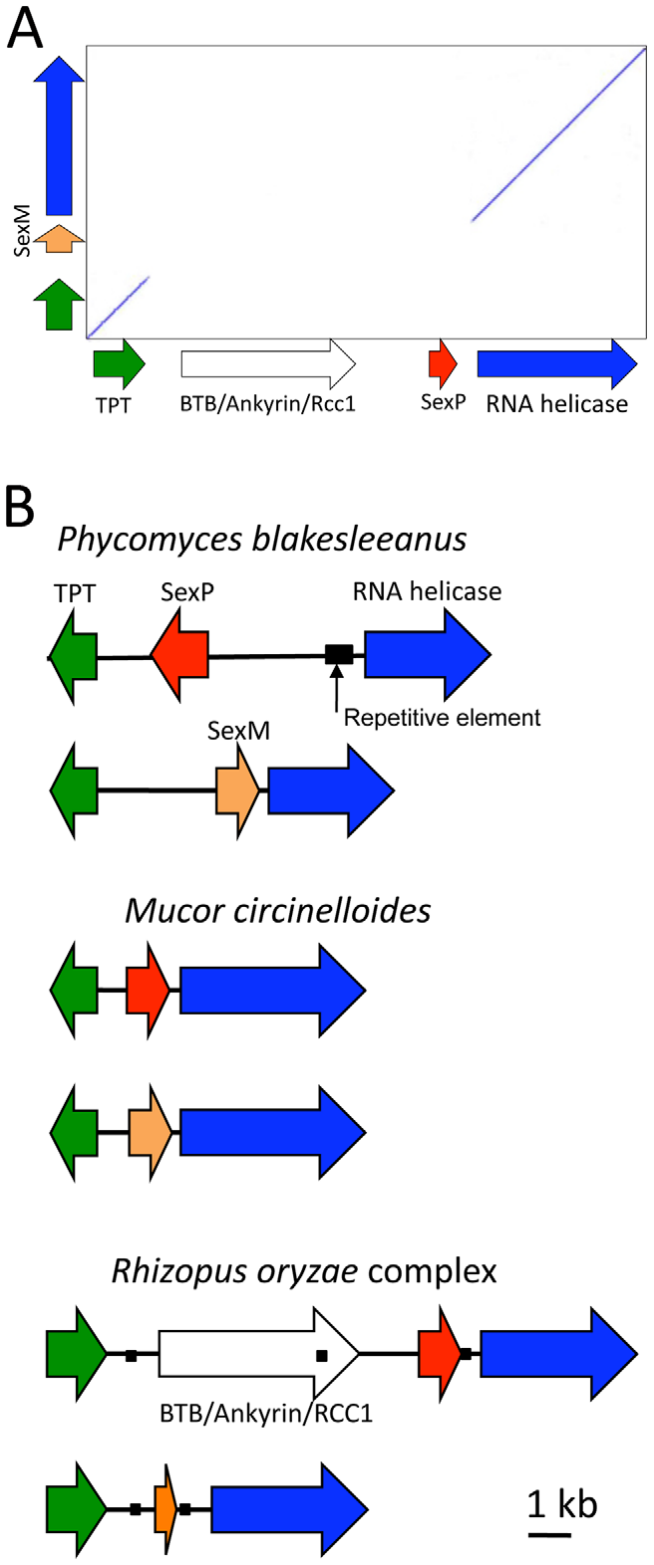
Comparison of *sex* locus structure in the Mucorales (Mucoromycotina, Zygomycota). (A) Dot plot showing the divergent DNA sequence of *sexP* and *sexM* genes in *Rhizopus oryzae*, whereas the TPT and RNA helicase genes are highly conserved. The orientations of *sexP* and *sexM* genes are the same in *R. oryzae*. (B) *sex* locus structures of *P. blakesleeanus* and *M. circinelloides* [46] are compared with *R. oryzae* (this study). Relative to *R. oryzae*, the TPT gene in *M. circinelloides* and both the TPT gene and *sexP* in *P. blakesleeanus* have an inverted orientation. The (+) allele of *R. oryzae* contains an additional gene between the TPT gene and *sexP* that is not found in the other two species or in the (−) allele of *R. oryzae*. Black boxes show repetitive sequence tracts, arrows show gene orientation, and lines represent intergenic sequence.

Recent studies demonstrated that *P. blakesleeanus* and *M. circinelloides* have similar *sex* genes [16], [27]. The *sex* locus and the flanking genes in both species consists of a 7–10 kb region that includes a gene encoding a high mobility protein (HMG) that is flanked by a gene encoding a TP transporter (TPT) and a second gene coding for an RNA helicase. DNA sequences of *P. blakesleeanus* and *M. circinelloides* were used to screen the publicly available genome of *R. oryzae* and identify the *sex* locus in *R. oryzae* [16]. The *sex* locus of *R. oryzae* is located in a 13 kb region in Supercontig 1 (*R. oryzae* database, http://www.broadinstitute.org/annotation/genome/rhizopus_oryzae/MultiHome.html). It encodes proteins with 48% and 58% total amino acid identity with the (+) *sex* loci of *P. blakesleeanus* and *M. circinelloides*, respectively. Unlike *P. blakesleeanus* and *M. circinelloides*, which have *sex* and TPT genes in an inverted orientation [16], [27], the *sex* and flanking genes of both (+) and (−) strains of *R. oryzae* are oriented in the same direction (Fig. 2).

We designed specific PCR primers to amplify the *sex* locus from five representative strains of *R. oryzae*; one of which contained the putative (+) mating type and four strains with the putative (−) mating type. DNA sequencing confirmed that the mating locus structure of *R. oryzae* is similar to those of *P. blakesleeanus* and *M. circinelloides* and consists of an HMG gene (*SexP*/*SexM*) flanked by TP transporter and RNA helicase genes (Table 2, Fig. 2). The mating genes (HMG, TPT, and RNA helicase) of the (+) strains of these three species share 24% amino acid identity (49% similarity at the DNA level). The (−) alleles of these three species share 30% amino acid identity (92% similarity at the DNA level).

**Table 2:** Comparison of the number of genes, the length of individual genes, and the length of the entire gene complex in the *sex* loci of *P. blakesleeanus, M. circinelloides* and *R. oryzae* complex.

Parts of mating type locus	*P. blakesleeanus*		*M. circinelloides*		*R. oryzae*	
	(+)	(−)	(+)	(−)	(+)	(−)
GenBank accession #	EU009462	EU009461	FJ009107	FJ009106	HQ450315	HQ450316
Total fragment length^3^	9.97	7.63	6.88	6.86	13.31	6.96
Intergenic regions, total length^3^	5.33	3.53	0.57	0.88	3.21	1.5
TPT (EamA-like transporter family)^4^	289^1^		276^1^		440	
BTB domain & ankyrin repeat (RO3G_01289)^4^	-		-		395^2^	
BTB/POZ domain (RO3G_01289)^4^	-		-		590^2^	
Predicted protein, Rcc1 (RO3G_01290) 4	-		-		152^2^	
HMG (*MATA* box)^4^	258^1^	211	326	249	314	188
RNA helicase (RO3G_01291) 4	649		1231		1187	
Repetitive elements	1	-	-	-	3	2
Inverted repeats	-	-	-	-	1	-
Palindromes	-	-	-	-	3	1

1 Inverted genes.

2 Single ORF.

3 Length of DNA shown in kilobases.

4 Length of protein shown in amino acids.

The RNA helicase gene is 4161–4191 bp long and includes nine introns of 40–150 bp each. This gene was annotated in the *R. oryzae* database as transcript RO3G_01291 in Supercontig 1. The TP transporter gene, which is not yet annotated in the *R. oryzae* database, is 1336 bp long and belongs to the EamA-like transporter family. We also identified the putative protein-coding gene [16] with an ORF that was located between the TPT gene and the HMG gene in both the (+) genome strain RA99-880 and the (+) tester strain CBS346.36. Parts of this gene are annotated in the *R. oryzae* database as transcripts RO3G_01290 (1482 bp), RO3G_01289 (1831 bp), and RO3G_01288 (625 bp). These transcripts were identified as an Ankyrin repeat region, a BTB/POZ region, and a third protein of unknown function, respectively. This three-part region BTB/Ankyrin/RCC1 (Fig. 2) [16] is absent in all of the (−) strains that we tested as well as the publicly available genomes of *Phycomyces blakesleeanus* and *Mucor circinelloides* [16], [27]. The presence of BTB/Ankyrin/RCC1 accounts for the fact that the *sex* locus and flanking gene complexes from (−) strains are smaller (7 kb) than in the (+) strains (13.3 kb). Aside from BTB/Ankyrin/RCC1, the structure and direction of the flanking genes is similar in (−) and (+) strains.

Strain RA99-880, used to obtain the genome sequence of *R. oryzae* [32], did not mate with any of the isolates that we tested. This observation led us to question whether this isolate represents *R. oryzae* or a different *Rhizopus* species (Table 3). We compared the (+) mating alleles of RA99-880 to CBS346.36. We previously determined that CBS346.36 produced zygospores when mated with the holotype culture of *R. oryzae* CBS112.07, suggesting that they are likely conspecific. We determined that the HMG box region in the (+) strain CBS346.36 was 942 bp long, contained no introns, and shared only 93% DNA sequence identity with genome strain RA99-880.

**Table 3:** Comparison of the phylogenetic signal obtained from different genes using different methods for delimitation of the two cryptic species, *Rhizopus oryzae s. s*. and *Rhizopus delemar*; percent difference is depicted for both DNA (bp) and amino acids (aa).

Locus	Length (bp)	% Difference (bp)	% Difference (aa)	ML Bootstrap Support
mtSSU	370	-	n. a.	-
rDNA 28S	950	0.3	n. a.	-
rDNA ITS1-5.8S-ITS2	526	1.7	n. a.	83
*RPB2*	759	3	4.7	99
HMG (+)	466	3.7	4.5	100
HMG (−)	632	3.8	6.2	99
TPT	978	4.1	5.5	100
RNA helicase	764	5.1	7.2	100

In addition, we compared the structures of the HMG box region between (−) and (+) alleles. The (−) allele encodes a protein that is 180 amino acids in length whereas the (+) allele encodes a protein that is 253 amino acids in length. Both (+) and (−) alleles share 23% nucleotide identity but both contain an identical amino acid motif at the 5′-end of their sequences: RP(T)NAF(I)LY. This motif is nearly identical to the motifs found in both the (+) and (−) strains of *Phycomyces blakesleeanus* and *Mucor circinelloides.*

### Multilocus phylogenetic studies

Comparative analysis of mating loci between two (+) and four (−) strains of *R. oryzae* revealed that two (+) strains, CBS346.36 and RA99-880, shared 93.3% amino acid identity. Although the DNA sequences from (−) strains were more conserved, we still detected significant differences between isolates: these four strains shared 98.4–100% amino acid identity. This genetic divergence between isolates of the same mating type prompted analysis of genetic diversity among multiple isolates of *R. oryzae*.

**Figure 3:**
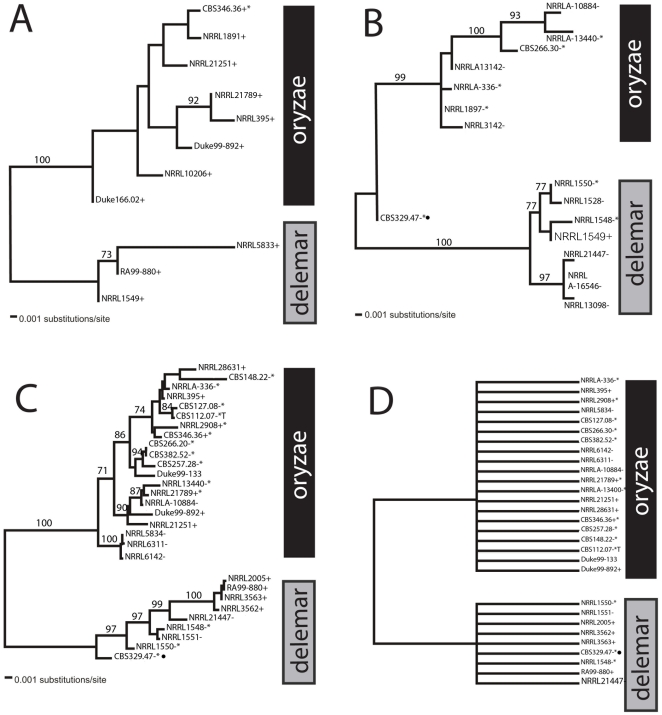
Maximum Likelihood phylogenies of the *Rhizopus oryzae* complex clearly delimit the two cryptic species, *Rhizopus oryzae s. s.* and *Rhizopus delemar*. (A) *sexP* alleles, (B) *sexM* alleles, (C) concatenated phylogram of rDNA ITS1-5.8S-ITS2, *RPB2*, TPT gene, and RNA helicase gene, and (D) four-gene strict consensus tree. Analysis included a total of 458 (+), 635 (−) and 3064 (MLS) nucleotide characters. ML bootstrap proportions higher than 70 are shown above the nodes. Asterisks (*) indicate strains that produced zygospores. Filled circles indicate a single *R. delemar* strain, CBS329.47, closest to the ancestral state.

To determine genetic relationships among isolates, we obtained partial sequences of the HMG gene from 57 strains using PCR primers specific for (+) and (−) mating alleles. Based on their DNA sequences, 26 strains belonged to the (+) type and 29 strains belonged to the (−) type (Table S3). These partial sequences were used for determining phylogenetic relationships among the strains. Eleven haplotypes of the (+) HMG locus were identified, which were separated into two distinct groups on the ML tree (Fig. 3A). Fifteen different haplotypes of the (−) HMG locus were identified, which also were separated into two well-supported phylogenetic groups (Fig. 3B). We also obtained partial sequences from the two flanking genes, TPT and RNA helicase. Eighteen TPT and 16 RNA helicase haplotypes were detected among the 41 strains. Gene trees for each locus strongly support two clades (Figs. S1C, D). Four additional gene regions commonly used in fungal systematics (mitochondrial small-subunit RNA (mtSSU), nuclear-encoded large subunit RNA (nLSU 28S), ITS1-5.8S-ITS2 region and *RPB2*) were also sequenced from the complete set of 57 clinical and environmental strains (Table S3). MtSSU and rDNA 28S genes were too highly conserved to provide phylogenetic resolution within the *R. oryzae* complex (data not shown). In contrast, phylogenetic analysis of the more variable rDNA ITS1-5.8S-ITS2 and *RPB2* regions revealed the same two well-supported clades detected by the analysis of the mating loci. Phylogenetic analysis of the concatenated data set containing all four loci (rDNA ITS1-5.8S-ITS2, *RPB2*, TPT and RNA helicase) also strongly supports two major clades (Fig. 3C, also Fig. S1A). These two clades correspond to two cryptic species, *Rhizopus oryzae sensu stricto* and *R. delemar*, that had been previously recognized based on *LDH* genes and ITS sequencing [9].

Although separate and combined gene trees all support evidence for two cryptic species, phylogenetic relationships among strains within each species varied depending on which gene was used. This is also illustrated by the failure of consensus trees to resolve phylogenetic relationships within either cryptic species (Fig. 3D). A phylogenetic congruence test [33] revealed significant discordance among gene phylogenies attributable to within-species recombination among all 4 loci (partition homogeneity test, p = 0.001). This scenario, whereby multiple gene trees consistently resolve the phylogeny between but not within species, is the basis for the Phylogenetic Concordance Species Concept advocated by Taylor *et al*. [34].

When we mapped results of the mating experiments to the ITS+*RPB2*+TPT+RNA helicase phylogeny (Fig. 3C) we determined that many isolates of *R. oryzae s. s.* were capable of producing zygospores when paired with isolates of the opposite mating type. Specifically, three (+) and eight (−) strains out of 33 *R. oryzae s. s.* isolates (11/33 or 33%) were capable of a successful mating reaction as defined by the production of zygospores. In contrast, most *R. delemar* strains were sterile. Only three of 21 *R. delemar* strains were capable of producing zygospores when paired with the (+) tester strain CBS346.36 (3/21 or 14%). However, none of the strains produced zygospores when paired with the (+) genome strain RA99-880 from the same clade.

### Distribution of mating types within each cryptic species

To test if the distribution of the two mating types was the same in populations of *R. oryzae* and *R. delemar,* we used mating gene-specific PCR primers to assign mating types to 57 different strains. All strains possessed either a (+) or a (−) HMG mating allele (mating allele type could not be determined for two strains). As expected, (+) strains were only capable of mating with (−) strains and *vice versa*. In each species, the ratio of plus/minus mating types is close to 1∶1, evidence that is consistent with a randomly mating population. Within *R. oryzae*, the ratio of +/− isolates was observed to be 14∶18 (chi-square with one degree freedom  = 0.5, two tailed p = 0.4795). For *R. delemar* this ratio of plus/minus was 12∶11 (chi-square = 0.043, p = 0.8348). For all isolates combined (regardless of species) this ratio (26∶29) is also not significantly different from 50∶50 (chi-square = 0.164, p = 0.6568).

### Asexual sporangiospores of *R. oryzae* and *R. delemar* are morphologically indistinguishable

When grown on agar media, all *R. oryzae* complex strains have similar growth patterns and growth rates. Actively growing colonies fill a 90 mm Petri dish in 2 to 3 days at room temperature (23°C). After 3 to 4 days, most isolates begin to produce sporangiospores (Fig. S2B), although some strains sporulate more slowly and may require 10 to 14 days.

**Figure 4:**
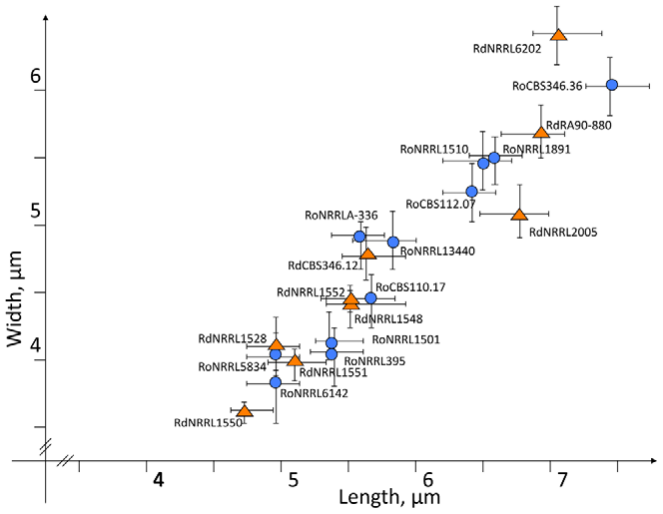
Range of sporangiospore sizes for isolates from the *Rhizopus oryzae* complex. Naming convention includes the species prefix (Ro – *Rhizopus oryzae*, Rd – *Rhizopus delemar*) followed by the strain number. Bars indicate the 95% confidence interval.

**Figure 5:**
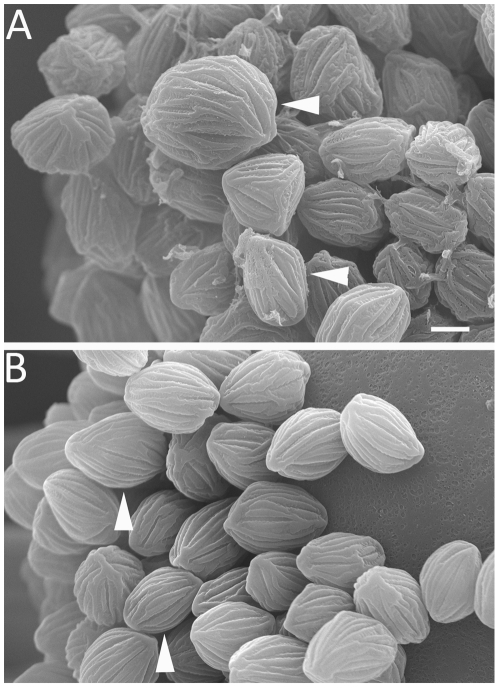
Main pattern of asexual sporangiospore micromorphology. (A) *Rhizopus delemar* NRRL3562. (B) *R. oryzae* NRRL2908. Arrows show the variation in spore size on the same sporangium (×5000). Scale bar = 1 µm.

In an effort to delimit *R. oryzae* from *R. delemar* isolates based on morphology, we measured the length and width of sporangiospores from 20 randomly chosen strains. The *R. oryzae* and *R. delemar* isolates we studied showed no observable differences in spore size (Fig. 4). Size differs considerably even for the spores produced by the same sporangium (Fig. 5), which reflects the different number of nuclei per spore. Binucleate sporangiospores are considerably larger than uninucleate ones (Fig. S2D). Our findings agree with previous studies [8] that both *R. oryzae* and *R. delemar* can have a wide and overlapping range of spore sizes. The length to width ratio remains approximately constant at 1.3.

Spore surface area was strain-specific for each of 12 randomly selected *R. oryzae* complex isolates (Fig. S3). However, we were unable to determine a specific pattern distinguishing sporangiospores of *R. oryzae* from *R. delemar*.

### *Rhizopus oryzae s. s.* and *R. delemar* strains can be differentiated by PCR with primers specific to lactate dehydrogenase (*LDH*) genes

**Figure 6:**
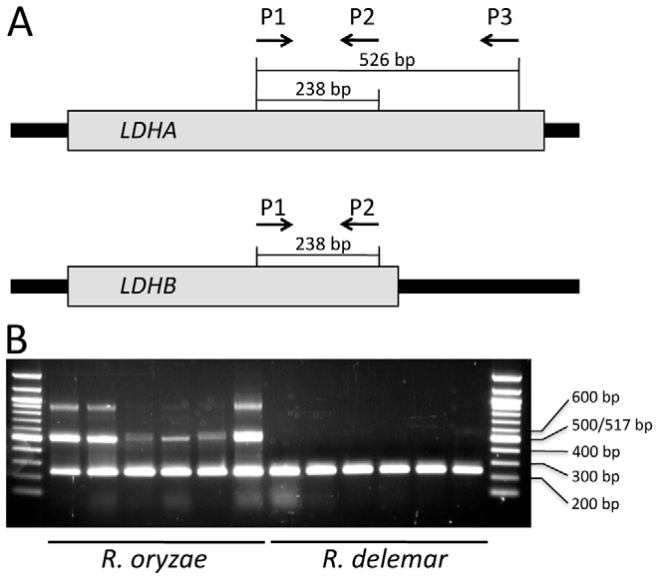
Different copy numbers of lactate dehydrogenase (LDH) in *Rhizopus oryzae s. s.* and *Rhizopus delemar*. (A) *LHDA* has a longer the 3′ end compared to *LDHB*. Primers P1 and P2 recognize both *LDHA* and *LDHB* producing 238 bp DNA fragments in PCR, whereas P3 only recognizes the 3′ end of the *LDHA* producing 525 bp DNA fragments in PCR with P1. Gene sizes are not to scale. (B) *Rhizopus oryzae s. s.* strains produces 238 bp and 525 bp bands in a PCR reaction with the three primers P1, P2, and P3, however, *R. delemar* strains produce only 238 bp bands with the same PCR conditions. Non-specific amplification of 900 bp bands occurred with several *R. oryzae s. s.* strains. Size marker is 100 bp ladder (NEB, Ipswich, MA, USA). *Rhizopus oryzae s. s.* strains are (from left to right) NRRL2908, CBS346.36, NRRLA-336, CBS110.17, NRRL1897, NRRL1501 and *Rhizopus delemar* strains are (from left to right) ATCC34612, NRRL2871, NRRL2005, NRRL1528, NRRL21447, NRRL13908.

Abe et al. [10] demonstrated that *R. oryzae* contains two copies of *LDH*, *LDHA* and *LDHB*, whereas *R. delemar* has only a single copy of this gene. We developed PCR primers that amplify portions of the *LDHA* and *LDHB* genes and tested them on 12 isolates from the *R. oryzae* complex, including six randomly selected isolates each of *R. oryzae s. s*. and *R. delemar* (Fig. 6A). All of the *R. oryzae s. s.* isolates produced an amplification product for both *LDHA* (ca 500 bp) and *LDHB* (ca 230 bp). In some cases, a nonspecific, higher molecular weight PCR product was also apparent. Sequencing of PCR products of *R. delemar* isolates confirmed it as the expected portion of *LDHB*. For *R. delemar* only *LDHB* products were amplified (Fig. 6B).

## Discussion

### Structure of the *sex* type locus in the *R. oryzae* complex

We examined the structure of the putative *sex* type locus in six representative strains of *R. oryzae* and demonstrate that *R. ozyzae* possesses a *sex* type locus that is homologous to those of other zygomycetes and also of microsporidians. This gene cluster consists of an HMG domain gene flanked by RNA helicase and TP transporter genes [16]. The characterization of *R. oryzae sex* genes constitutes the third representative of the Mucoromycotina (Zygomycota) for which the mating gene structure is known. When compared with the recently described *sex* loci of *P. blakesleeanus* and *M. circinelloides*, the (+) allele of the *R. oryzae sex* locus was significantly larger due to the presence of an additional putative ORF. However, this inserted gene is absent in the (−) allele of the locus. Unlike the *sex* locus of *P. blakesleeanus* and *M. circinelloides*, which contain TPT and HMG genes in inverted orientations, all genes in the *R. oryzae sex* type locus were positioned in the same orientation (Fig. 2). The overall similarity between the *sex* type locus of *R. oryzae* and other zygomycetes was significant, suggesting that this locus is involved in regulation of the mating process [28]. Further functional genetic analysis is necessary to confirm this hypothesis.

### Cryptic speciation in the *Rhizopus oryzae* complex

Our phylogenetic analyses confirm the previous observations by Abe *et al.* [10], [35] that *R. oryzae s. l.* can be subdivided into two cryptic species groups, designated *R. oryzae s. s.* and *R. delemar.* We confirmed that the genome strain RA99-880, once thought to belong to *R. oryzae s. s.*, is actually a member of *R. delemar* [32]. Genetic isolation between these two cryptic species was evident in the strongly supported four-gene phylogeny and the genealogies of both the (+) and (−) alleles of the HMG genes (Fig. 3). The higher number of amino acid and nucleotide changes in the *sex* genes relative to the “standard” genes (Table 3) suggests that the *sex* genes may prove useful in resolving phylogenetic questions in other zygomycetes and perhaps in other fungi.

Saito *et al*. [14] were also able to distinguish *R. oryzae s. s.* and *R. delemar* based on rDNA phylogeny and physiological differences in acid production. They confirmed the presence of two distinctive physiological groups: isolates that produce lactic acid and have two copies of the *LDH* gene (*R. oryzae s. s.*) and isolates that produce both fumaric and malic acids but have only *LDHB* (*R. delemar*). We designed primers for the *LDH* genes and confirmed a similar pattern in the 57 isolates we studied. It has been suggested that *R. oryzae'*s ability to ferment various organic substrates may explain why this species was domesticated and used to process various types of Asian fermented foods [4], [11]. Lactate dehydrogenases (LDH) are important for these enzymatic processes [2], [4], [11], [36], [37].

Despite the fact that *R. oryzae s. s.* and *R. delemar* can be resolved by multi-gene phylogenetic analyses and physiological differences in acid production, morphological examinations and molecular analyses of more conservative loci indicate that these two cryptic species are very close relatives. For example, sequences of the mtSSU and 28S rDNA genes were too highly conserved to provide any phylogenetic resolution within the *R. oryzae* complex. Similarly, analyses of secondary structure in the ITS2 region indicates that there are no complementary base pair changes (CBC) between *R. oryzae s. s.* and *R. delemar,* whereas other species in the genus *Rhizopus* are quite different (Table 4). Furthermore, isolates of *R. oryzae s. s.* and *R. delemar* exhibited similar colony morphology, growth characteristics, and spore morphology. Although sporangiospore size is of foremost importance in the classification of the genus *Rhizopus* [38], [39], spore size measurements were insufficient to distinguish *R. oryzae* from *R. delemar*.

**Table 4:** Analysis of similarity between *Rhizopus oryzae s. s.* and other *Rhizopus* species in ribosomal DNA sequences (ITS1-5.8S-ITS2, 28S) and predicted compensatory base changes (CBC) for the ITS2 region.

	rDNA ITS1-5.8S-ITS2	rDNA 28S	CBC
*R. delemar*	98.3% (HQ435103)	99.7% (HQ435039)	0
*R. microsporus*	65% (HQ450314)	95% (HQ435046)	2
*R. homothallicus*	68% (EU491016)	94% (DQ641324)	2
*R. azygosporus*	43% (DQ641314)	93% (DQ466597)	2
*R. caespitosus*	43% (DQ641325)	93% (DQ466604)	2
*R. schipperae*	43% (DQ641323)	92% (DQ466606)	2
*R. sexualis*	55% (DQ641322)	89% (DQ466592)	8
*R. stolonifer*	39% (FN401529)	89% (DQ466595)	6

### Sexual reproduction of *R. oryzae* and other zygomycetes in the laboratory and in nature

Fungi employ a variety of reproductive modes, in the lab as well as in nature. Whereas some fungal species appear to be obligate sexual organisms (e.g. [40], [41]) and others appear to be strictly asexual [42], [43], [44], the majority of fungi apparently have a mixed mating system that includes both sexual and asexual reproduction (e.g. [45], [46], [47]). Although earlier studies suggested a predominantly asexual mode of reproduction in *R. oryzae* [3], [4], analysis of sequence variation in multiple loci suggests that this species complex comprises two cryptic species and both exhibit a sexual mode of reproduction.

Zygospore formation is considered a hallmark of sexual reproduction. However, only 10% of described Mucoromycotina species are known to produce zygospores, either under natural conditions or in pure culture [48], [49]. Despite relatively frequent formation of zygospores in closely related heterothallic (*R. stolonifer, R. microsporus*) and homothallic (*R. sexualis, R. homothallicus*) species, only a few reports have described zygospore production between isolates of the *R. oryzae* complex [3], [4], [21], [25], [26]. In our study, 40% of the isolates of *R. oryzae s. s.* were capable of producing zygospores in the laboratory when paired with isolates of the opposite mating type. The ability to produce zygospores with a suitable mating partner seems to be characteristic of almost all subgroups of the *R. oryzae s. s.* clade (Fig. 3).

Most strains producing zygospores in our study belong to the *R. oryzae s. s.* clade (Table S3). Although most isolates of *R. oryzae s. s*. were capable of producing zygospores, we observed that their morphology was more similar to the azygospores observed in other zygomycetes. Zygospores of *R. oryzae* possess asymmetric suspensors of unequal size, a feature associated with azygospore production in *R. stolonifer* and *R. azygosporus* [3], [6], [18], [19]. Single asymmetric suspensors have been reported in different varieties of *R. microsporus* [4], and in distantly related species from the genera *Absidia* and *Zygorhynchus* [50]. In the genus *Rhizopus*, zygospore formation may not be a useful character for delimiting species boundaries because many *Rhizopus* isolates form zygospores even when their mating partner belongs to a different species [51].

In our study, *R. oryzae* zygospores did not germinate into germosporangia [52] under any of the tested experimental conditions. When zygospores were mechanically disrupted, the multinuclear protoplast developed into a vegetative mycelium that contained nuclei of only the (−) mating type, indicating that meiosis did not occur. If meiosis had occurred, we would instead expect equal proportions of (+) and (−) mating types. This result suggests that mating and recombination might not occur under laboratory conditions.

In contrast, zygospores of the closely related species *R. stolonifer* can germinate in two different ways. When zygospores are geminated prior to maturation, they form a vegetative mycelium that is either (+) or (−). When mature zygospores are germinated, they form a germosporangium that produces spores with equal proportions of (+) and (−) mating types [25], [26], [49]. Germination of zygospores into vegetative mycelia is also known in distantly related species in the genus *Zygorhynchus* [21].

Although we did not directly detect successful mating and progeny development in the laboratory, we did observe nearly equal frequency of (+) and (−) mating types across both cryptic species within the *R. oryzae* species complex (Table S3). This provides strong indirect evidence for sexual recombination in *R. oryzae.* Analysis of multiple gene phylogenies provides further evidence for outcrossing. Phylogenetic analyses of individual and combined loci consistently support two well-defined sibling species, *R. oryzae s. s.* and *R. delemar*. Within each species, however, discordant gene trees suggest that both species are inherently sexual, with an outcrossing mode of reproduction (Fig. S1, supplementary materials). If populations of each species were clonal, we would expect independent gene trees to be concordant [33]. In this study, we consistently observed conflict between gene partitions within both species (ph test, p<.001), suggesting recombination between loci. Together with equal distribution of sexes, our results strongly support the hypothesis that genetic recombination is occurring within both *R. oryzae s. s.* and *R. delemar*. Further studies employing population-level sampling, along with genome sequencing of *R. oryzae s. s.* will be necessary to determine the extent and nature of recombination in both species.

Mucormycosis is an emerging infectious disease [53] and is increasingly reported as a cause of fungal infection in patients with impaired immunity [17]. Isolates from the *R. oryzae* complex are the most common zygomycetes infecting humans [54], [55]. Our phylogenetic studies also suggest that the two cryptic species, *R. delemar* and *R. oryzae,* may differ in relative virulence. It is also possible that *R. delemar* and *R. oryzae* may differ in susceptibility to antifungal drugs [55], [56]. In the pathogenic basidiomycete, *Cryptococcus neoformans*, the *MAT* locus is linked to virulence [57], [58]. It is possible that the *sex* locus might be similarly involved in the pathogenesis of *Rhizopus* species.

### Conclusion

Our data support the hypothesis that the genetic machinery for sexual reproduction is conserved among multiple genera of Mucoromycotina. *Rhizopus oryzae* is one of the most economically important members of this group. Our multi-gene phylogenetic analyses support the existence of two cryptic species: *R. delemar* and *R. oryzae*. Notably, the recently sequenced genome isolate is a member of *R. delemar,* so it would be ideal to also sequence a representative genome of *R. oryzae s. s*. We demonstrate that both species have the potential for sexual reproduction, and although our mating studies suggest that sexual reproduction is infrequent, strains of both mating types are equally abundant in both species.

## Materials and Methods

### Cultivation of fungal strains

Strains of *Rhizopus oryzae s. l.* were obtained from different culture collections: American Type Culture Collection (ATCC), Duke University, Fungal Genetics Stock Center (FGSC), Agricultural Research Service Culture Collection (NRRL) in USA, Centraalbureau voor Schimmelcultures (CBS) in the Netherlands and Friedrich-Schiller University Fungal Reference Center in Germany (Table S3). Replicate isolates of several mating type tester strains first described for *R. oryzae* by Schipper [3] were included. Strains were grown on 10% Potato-Dextrose Agar (PDA) (NEOGEN, Lansing, MI, USA), in tubes and 90 mm Petri plates at room temperature.

### Light microscopy and fungal morphology

Wet mounts of asexually sporulating isolates were examined at 100–400× under an Olympus CX31 light microscope equipped with an ocular micrometer (Olympus America Inc., Center Valley, PA, USA). To test whether morphology could be used to differentiate genetically related strains within the *R. oryzae* complex we made 100 measurements of sporangiospore width and length from 20 randomly chosen strains (Fig. 4). We then analyzed the morphology and size of *R. oryzae s. l.* spores using multivariate discriminate analysis and cluster analysis [59], [60], [61]. The following statistical parameters were examined for asexual sporangiospore dimensions: average (*M*), variance (*D*), standard deviation (*σ*), and standard error (±*m*). All statistical analyses were conducted in the STATISTICA 6.0 software package (StatSoft Inc., Tulsa, OK, USA).

### Mating assays

Mating tests were conducted in 4.5 or 9 cm diameter Petri plates with several types of standard agar growth media: PDA, Malt Agar (MA) (VWR International, Bristol, CT, USA), Glucose Peptone Yeast Extract Agar (GPYA) (HiMedia Laboratories Pvt. Ltd., Mumbai, India), Sabouraud agar [62], and water agar [52]. Mating tests were conducted at three different nutrient levels; 10%, 50%, or 100% of the level recommended by the manufacturer to test the affect of nutrients on zygospore germination. Petri plates were either inoculated with 1×1 cm agar pieces taken from the growing edge of the colony, or with a 20 µl suspension of lyophilized tissue. In each mating test inocula from two isolates were placed 1 cm from each other on a fresh Petri plate. Cultures were then incubated in either complete darkness or exposed to light and they were either sealed or not sealed with Parafilm (Pechiney Plastic Packaging, Menasha, WI, USA) at room temperature according to Hesseltine and Rogers [29]. Development of conjugating hyphae and zygospores was observed at 100–400× under an Olympus CX31 light microscope (Olympus America Inc., Center Valley PA, USA) or at 200–3500× with a JSM-5900LV scanning electron microscope (SEM) (JEOL U.S.A., Peabody MA, USA). Observations for mating were conducted several times each week for a period of 6–8 weeks. For mating crosses that produced zygospores, we attempted to stimulate germination following traditional protocols [29]. We also tested whether it was possible to grow ungerminated zygospores by crushing them with forceps and then incubating them on nutrient agar.

### DNA extraction, amplification and sequencing

Prior to DNA isolation cultures were incubated for 8 to 20 hours at room temperature in 50 ml of Potato Dextrose broth (NEOGEN, Lansing, MI, USA) and were then filtered through a sterilized Mira cloth [36]. Mycelium was then lyophilized for 1 to 2 days and ground in liquid nitrogen with a mortar and pestle or glass beads.

DNA was extracted following the CTAB extraction technique [63]. PCR mixtures with a total volume of 25 µl per sample were prepared according to the protocol supplied by TaKaRa (TaKaRa Bio Inc., Otsu, Shiga, Japan). PCR was performed according to the basic protocols outlined by White *et al*. [64] for all genes except where specifically noted below. The internal transcribed spacer region of rDNA (ITS1-5.8S-ITS2) was amplified with primers ITS1 and ITS4 [64]. Partial large subunit rDNA 28S was amplified using the primers LROR [65] and LR5 [66]. The mitochondrial SSU was amplified with the primer set mtSSU1_f and mtSSU2_r [67]. The genome of *R. oryzae* has two non-identical copies of *RPB2* located on different supercontigs. Accordingly, it was not possible to use the coding domains 5–7, which are located in the central part of the gene and have traditionally been used in fungal phylogenetics. To overcome this problem, we obtained DNA sequences of coding domains 1–3, which are unique to the *RPB2* copy located on Supercontig 10. To amplify the 5′ end (domain 1, intron 1 and part of domain 2) of the RNA Polymerase II subunit 2 gene (*RPB2*), we designed three new primers (Rh_RPB2_f, Rh_RPB2l_r and Rh_RPB2s_r – Table S1) using the *RPB2* sequence from the *R. oryzae* genome database. For *RPB2* a “touchdown” PCR protocol was used as described by Don *et al*. [68].

All PCR-amplified fragments were separated by electrophoresis on a 1.5% agarose gel stained with SYBR® Safe and visualized with a UV transilluminator. PCR products were purified with Qiagen Quick-Clean columns (Qiagen Inc., Valencia, CA, USA) or with ExoAP enzymes [69]. PCR products were then sequenced using amplification primers and Big Dye chemistry version 3.1 (Applied Biosystems Inc., Foster City, CA, USA), and the DNA sequences were run on an ABI3700 DNA sequencer (Applied Biosystems Inc., Foster City, CA). DNA sequences generated for this study were edited in Sequencher 4.0 (Gene Codes, Ann Arbor, MI, USA), and are available in GenBank (Table S2).

### Phylogenetic analysis

We characterized rDNA 28S and ITS1-5.8S-ITS2, mtSSU, HMG, *RPB2*, TPT, and RNA helicase genes for 41 isolates (Table 3). We also examined the phylogenetic pattern of the “plus” and “minus” mating alleles to see whether these regions could be used to distinguish between different lineages from the *R. oryzae* complex. First, we compared the phylogenetic signal of the “plus” and “minus” alleles to other genes that have traditionally been used for phylogenetic analyses of fungi (rDNA 28S and ITS1-5.8S-ITS2, *RPB2*). Next, we compared the phylogenetic signal of the “plus” allele with the “minus” allele to see if inferences from each of these alleles were similar and which region was more informative. Phylogenetic relationships were determined by Maximum Likelihood (ML) and phylogenetic support was assessed for each gene by bootstrap analyses (ML) using PAUP* 4.0a109 [70]. Using sequences of rDNA ITS1-5.8S-ITS2, *RPB2*, TPT, and the RNA helicase genes, the maximum likelihood approach was applied to generate a four genes tree for all of the 41 studied isolates. The partition homogeneity test was run on PAUP* 4.0a109 using a parsimony criterion for four genes and 1000 replicates [70].

After characterizing the *sex* genes of several *R. oryzae* isolates, we compared them to the previously characterized *sex* alleles of *Phycomyces blakesleeanus* (EU009461, EU009462) and *Mucor circinelloides* (FJ009106, FJ009107). Nucleotide and amino acid similarity of HMG, TPT and RNA helicase gene/protein alignments was examined in Multalin [71] and ClustalW2 [72].

To generate a dataset of *Rhizopus* reference taxa, additional rDNA 28S and ITS1-5.8S-ITS2 sequences from identified *Rhizopus* species with accurate nomenclature were either downloaded from GenBank or generated in this study (Table 4) [73]. Compensatory base changes in the ITS2 region for *Rhizopus* species were calculated using the ITS2 Database and the programs 4Sale and CBCAnalyzer [74], [75], [76], [77]. We did not detect any compensatory base changes between *R. oryzae* and *R. delemar* in the ITS2 region (Table 4).

All sequences were initially aligned in ClustalX [78]. Alignments were manually adjusted and ambiguous regions were excluded from the alignments using Mesquite 2.5 [79]. *sex* loci and other genes of the strains of interest were aligned and compared using Sequencher 4.0 (Gene Codes, Ann Arbor, MI, USA), Multalin [71] and ClustalW2 [72].

Repetitive sequences in “plus” and “minus” alleles of the *sex* locus were determined manually and using the Tandem Repeats Finder [80]. Inverted and palindromic repeats were identified using tools available at the REPEATS, SECONDARY STRUCTURE & MELTING TEMPERATURE web site (http://emboss.bioinformatics.nl/cgi-bin/emboss/). Directionality of the genes in the *sex* loci was determined in ORF Finder (www.ncbi.nlm.nih.gov/gorf/) and displayed with a dot plot.

### Analysis of mating genes

Primers for the TPT and RNA helicase flanking genes were designed using Primer3 [81] from the *R. oryzae* database sequences. PCR reactions using these primers were conducted using the TaKaRa protocol (TaKaRa Bio Inc., Otsu, Shiga, Japan). Products were isolated and purified using a gel extraction kit (Qiagen GmbH, Hilden, Germany), cloned with a TOPO TA kit (Invitrogen, Carlsbad, CA, USA), and sequenced as described above. New primers were subsequently designed at the end of the newly sequenced region and the process was repeated by “primer walking” to obtain DNA sequences between the flanking genes. A total of fifty-seven primers were employed to sequence the entire length of the *Rhizopus sex* locus, including the flanking genes (Table S1). The entire *sex* locus and the two flanking regions were sequenced for five strains (Table 2) and these data were used to design primer sets for direct amplification of the mating loci: SgeneCORE_for and SgeneCORE_rev for the “minus” allele and Plus1_f and Plus1_r for the “plus” allele (Table S1). PCR with the SgeneCORE primer set produced a fragment of 780–800 bp whereas PCR with the Plus1 primer set produced a fragment of 465–480 bp. To avoid formation of secondary structures during amplification of intergenic regions of the *sex* locus, 1% DMSO was added to both PCR and sequencing mixtures [82].

### Use of the Lactate Dehydrogenase (*LDH*) gene to differentiate *R. oryzae sensu stricto* from *R. delemar*

Lactate dehydrogenase genes encode hydrolytic enzymes that enable *R. oryzae* to grow in decaying organic matter rich in complex carbohydrates [38]. Previous research has shown that differences in the *LDH* genes can differentiate *R. delemar* from *R. oryzae s. s. Rhizopus delemar* has only one copy of the gene (*LDHB*) whereas *R. oryzae s. s.* has two copies (*LDHA* and *LDHB*) [11], [14] (*Rhizopus oryzae* database). The sequences of these two genes are almost identical except that *LDHA* has a longer 3′ ORF. Although we did not include this locus in our phylogenetic analyses, we developed a PCR-based method to efficiently distinguish between *R. oryzae s. s*. and *R. delemar* using a combination of one forward and two reverse primers for the *LDH* gene. The forward primer JOHE22917 anneals to both genes. The reverse primer JOHE22918 anneals to both *LDHA* and *LDHB* whereas the other reverse primer JOHE22919 recognizes only *LDHA* (Fig. 6A). When PCR is performed with all three primers, *R. oryzae s. s.* isolates yield two fragments of different sizes: one PCR product for *LDHA* and another for *LDHB*. Isolates of *R. delemar* yield only one PCR product, however. We randomly selected six strains each of *R. oryzae s. s.* and *R. delemar* to test the *LDH* PCR assay. Results were compared with phylogenetic analyses used to distinguish these species.

## Supporting Information

Figure S1 Maximum Likelihood phylogeny for rDNA ITS1-5.8S-ITS2 (A), *RPB2* (B), TPT (C) and RNA helicase (D) genes. Analysis included a total of 566 (rDNA ITS1-5.8S-ITS2), 757 (*RPB2*), 978 (TPT) and 764 (RNA helicase) nucleotide characters. ML bootstrap proportions higher than 70 are shown above the nodes. Group * includes ITS sequences AB097299, AB181316-AB181330 of *Rhizopus delemar*; group ** includes the ITS sequences AB181303-AB181309, AB181311-AB181315, AB097334 of *Rhizopus oryzae* [10]. T – type culture of *R. oryzae s. s.*, T' indicates a strain with an rDNA ITS1-5.8S-ITS2 sequence that is identical to the type culture of *R. delemar* (CBS120.12).
(TIF)

Figure S2 Sexual (zygospores) and asexual (sporangiospores) spores of *Rhizopus oryzae*. (A) Electron micrograph of a cross between *R. oryzae* strains CBS346.36 × CBS110.17 showing zygospores (black arrow heads). Scale bar  =  50 µm. (B) Asexual sporangium of *R. delemar* strain NRRL3563 without sporangium wall. Scale bar  =  20 µm. (C) Germinating of zygospore's protoplast (white arrow) into vegetative mycelium after crushing of lateral spore wall (black arrows). Scale bar  =  50 µm. (D) Different size of uni- (black arrow) and binucleate (white arrow) DAPI stained sporangiospores of *Rhizopus delemar* RA99-880. Scale bar  =  20 µm.
(TIF)

Figure S3 Micromorphology of sporangiospores. Panel A) *Rhizopus delemar* (from top to bottom): ATCC34612, RA99-880, NRRL3562. Panel B) *Rhizopus oryzae* s. s.(from top to bottom): NRRL2908, Duke99-133, NRRL3142. Panel C) Germination of the *Rhizopus oryzae s. s.* strain, CBS112.07. Scale  =  1 µm for panel A and B, and 5 µm for panel C.
(TIF)

Table S1 List of primers designed in this study.
(DOC)

Table S2 GenBank accession numbers of the sequences generated in this study.
(DOC)

Table S3 Isolates of the *Rhizopus oryzae* complex and related species used in this study; isolates of *Rhizopus oryzae* and *Rhizopus delemar* are named according to their placement in phylogenetic trees (See Results).
(DOC)

## References

[1] Ellis JJ (1985). Species and varieties in the *Rhizopus arrhizus–Rhizopus oryzae* group as indicated by their DNA complementarity.. Mycologia.

[2] Liou GY, Chen CC, Yuan GF, Chien CY (2001). A taxonomic study of the genus *Rhizopus* by isosyme patterns.. Nova Hedwigia.

[3] Schipper MAA (1984). A revision of the genus *Rhizopus*. I. The *Rhizopus stolonifer*-group and *Rhizopus oryzae*.. Studies in Mycology.

[4] Zheng RY, Chen GQ, Huang H, Liu XY (2007). A monograph of *Rhizopus*.. Sydowia.

[5] Abdel-Hafez SII (1984). Composition of fungal flora of four cereal grains in Saudi Arabia.. Mycopathologia.

[6] Domsch KH, Gams W, Anderson T (1980). Compendium of soil fungi..

[7] Fisher A (1892). Phycomycetes: Mucorinae In Rabenhorst, Kryptogamen-Flora Deutschl Oest u d Schweiz.

[8] Mil'ko AA (1974). Identification guide to Mucoralean fungi..

[9] Abe A, Oda Y, Asano K, Sone T (2006). The molecular phylogeny of the genus *Rhizopus* based on rDNA sequences.. Bioscience, Biotechnology, and Biochemistry.

[10] Abe A, Sone T, Sujaya IN, Saito K, Oda Y (2003). rDNA ITS sequence of *Rhizopus oryzae*: its application to classification and identification of lactic acid producers.. Bioscience, Biotechnology, and Biochemistry.

[11] Kito H, Abe A, Sujaya I-N, Oda Y, Asano K (2009). Molecular characterization of the relationships among *Amylomyces rouxii*, *Rhizopus oryzae*, and *Rhizopus delemar*.. Bioscience Biotechnology Biochemistry.

[12] Oda Y, Yajima Y, Kinoshita M, Ohnishi M (2003). Differences of *Rhizopus oryzae* strains in organic acid synthesis and fatty acid composition.. Food Microbiology.

[13] Ogawa Y, Tokumasu S, Tubaki K (2004). An original habitat of tempeh molds.. Mycoscience.

[14] Saito K, Saito A, Ohnishi M, Oda Y (2004). Genetic diversity in *Rhizopus oryzae* strains as revealed by the sequence of lactate dehydrogenase genes.. Archives of Microbiology.

[15] Ainsworth GC, Austwick PK (1955). A survey of animal mycoses in Britain: mycological aspects.. Transactions of the British Mycological Society.

[16] Idnurm A, Walton FJ, Floyd A, Heitman J (2008). Identification of the *sex* genes in an early diverged fungus.. Nature.

[17] Ribes JA, Vanover-Sams CL, Baker DJ (2000). Zygomycetes in human disease.. Clinical Microbiology Reviews.

[18] Schipper MAA, Maslen MM, Hogg GG, Chow CW, Samson RA (1996). Human infection by *Rhizopus azygosporus* and the occurrence of azygospores in zygomycetes.. Journal of Medical and Veterinary Mycology.

[19] Scholer HJ, Muller E, Schipper MAA, Howard DH (1983). Mucorales.. Fungi pathogenic for humans and animals. A Biology.

[20] Schwarz P, Bretagne S, Gantier J-C, Garcia-Hermoso D, Lortholary O (2006). Molecular identification of zygomycetes from culture and experimentally infected tissues.. Journal of Clinical Microbiology.

[21] Cutter VM (1942). Nuclear behaviour in the Mucorales. II. The *Rhizopus*, *Phycomyces* and *Sporodinia* patterns.. Bulletin of the Torrey Botanical Club.

[22] Idnurm A, James TY, Vilgalys R, Heitman J, Kronstad JW, Taylor JW, Casselton LA (2007). Sex in the rest: mysterious mating in the Chytridiomycota and Zygomycota.. Sex in fungi.

[23] Hawker LE, Abbott PM, Gooday MA (1968). Internal changes in hyphae of *Rhizopus sexualis* (Smith) Callen and *Mucor hiemalis* Wehm. associated with zygospore formation.. Annals of botany.

[24] Blakeslee AF, Welch DS, Cartedge JL (1921). Technique in contrasting Mucors.. The Botanical Gazette.

[25] Gauger WL (1961). The germination of zygospores of *Rhizopus stolonifer*.. American Journal of Botany.

[26] Gauger WL (1977). Meiotic gene segregation in *Rhizopus stolonifer*.. Journal of General and Applied Microbiology.

[27] Lee S, Corradi N, Byrnes E, Torres-Martinez S, Dietrich F (2008). Microsporidia evolved from ancestral sexual fungi.. Current Biology.

[28] Lee SC, Ni M, Li W, Shertz C, Heitman J (2010). The evolution of sex: a perspective from the fungal kingdom.. Microbiology and Molecular Biology Reviews.

[29] Hesseltine CW, Rogers R (1987). Dark-period induction of zygospores in *Mucor*.. Mycologia.

[30] Blakeslee AF, Cartedge JL, Welch DS, Bergner AD (1927). Sexual dimorphism in Mucorales. I. Intraspecific reactions.. The Botanical Gazette.

[31] Hawker LE, Becket A (1971). Fine structure and development of the zygospore of *Rhizopus sexualis* (Smith) Callen.. Philosophical Transactions of the Royal Society, Biological Sciences.

[32] Ma L-J, Ibrahim AS, Skory C, Grabherr MG, Burger G (2009). Genomic analysis of the basal lineage fungus *Rhizopus oryzae* reveals a whole-genome duplication.. PLoS Genetics.

[33] Burt A, Carter DA, Koenig GL, White TJ, Taylor JW (1996). Molecular markers reveal cryptic sex in the human pathogen *Coccidioides immitis*.. Proceedings of the National Academy of Sciences of the United States of America.

[34] Taylor JW, Jacobson DJ, Kroken S, Kasuga T, Geiser DM (2000). Phylogenetic species recognition and species concepts in fungi.. Fungal Genetics and Biology.

[35] Abe A, Oda Y, Asano K, Sone T (2007). *Rhizopus delemar* is the proper name for *Rhizopus oryzae* fumaric-malic acid producers.. Mycologia.

[36] Skory CD, Ibrahim AS (2007). Native and modified lactate dehydrogenase expression in a fumaric acid producing isolate *Rhizopus oryzae* 99-880.. Current Genetics.

[37] Skory CD, Mertens JA, Rich JO (2008). Inhibition of *Rhizopus* lactate dehydrogenase by fructose 1,6-biphosphate.. Enzyme and Microbial Technology.

[38] Frye CB, Reinhardt DJ (1993). Characterization of groups of the zygomycete genus *Rhizopus*.. Mycopathologia.

[39] Inui T, Takeda Y, Iizuka H (1965). Taxonomic studies on genus *Rhizopus*.. Journal of General and Applied Microbiology.

[40] Fiore-Donno AM, Martin F (2001). Populations of ectomycorrhizal *Laccaria amethystina* and *Xerocomus* spp. show contrasting colonization patterns in a mixed forest.. New Phytologist.

[41] Xu JP, Sha T, Li YC, Zhao ZW, Yang ZL (2008). Recombination and genetic differentiation among natural populations of the ectomycorrhizal mushroom *Tricholoma matsutake* from southwestern China.. Molecular Ecology.

[42] Douhan GW, Rizzo DM (2005). Phylogenetic divergence in a local population of the ectomycorrhizal fungus *Cenococcum geophilum*.. New Phytologist.

[43] Faeth SH (2002). Are endophytic fungi defensive plant mutualists?. Oikos.

[44] Harrington TC, Rizzo DM, Worrall JJ (1999). Defining species in the fungi.. Structure and dynamics of fungal populations.

[45] Perez G, Slippers B, Wingfield BD, Hunter GC, Wingfield MJ (2010). Micro- and macrospatial scale analyses illustrates mixed mating strategies and extensive geneflow in populations of an invasive haploid pathogen.. Molecular Ecology.

[46] Ramirez-Prado JH, Moore GG, Horn BW, Carbone I (2008). Characterization and population analysis of the mating-type genes in *Aspergillus flavus* and *Aspergillus parasiticus*.. Fungal Genetics and Biology.

[47] Seidl V, Seibel C, Kubicek CP, Schmoll M (2009). Sexual development in the industrial workhorse *Trichoderma reesei*.. Proceedings of the National Academy of Sciences of the United States of America.

[48] Pidoplichko NM, Mil'ko AA (1971). Atlas of Mucoralean fungi..

[49] Gauger WL (1965). The germination of zygospores of *Mucor hiemalis*.. Mycologia.

[50] Hesseltine CW, Ellis JJ (1961). Notes on Mucorales, especially *Absidia*.. Mycologia.

[51] Schipper MAA, Gauger WL, Van Der Ende H (1985). Hybridization of *Rhizopus* species.. Journal of General Microbiology.

[52] Blakeslee AF (1904). Zygospore formation a sexual process.. Science.

[53] Brown J (2005). Zygomycosis: An emerging fungal infection.. American Journal of Health-System Pharmacy.

[54] Petrikkos GL (2008). Zygomycosis (Mucormycosis): an emerging or re-emerging disease?. Mycological Newsletter.

[55] Singh N, Aguado JM, Bonatti H, Forrest G, Gupta KL (2009). Zygomycosis in solid organ transplant recipients: a prospective, matched case-control study to assess risks for disease and outcome.. The Journal of Infectious Diseases.

[56] Ibrahim AS, Avanessian V, Spellberg B, Edwards JEJ (2003). Liposomal amphotericin B, and not amphotericin B deoxycholate, improves survival of diabetic mice infected with *Rhizopus oryzae*.. Antimicrobial Agents and Chemotherapy.

[57] Kwon-Chung KJ, Edman JC, Wickes BL (1992). Genetic association of mating types and virulence in *Cryptococcus neoformans*.. Infection and Immunity.

[58] Nielsen K, Marra RE, Hagen F, Boekhout T, Mitchell TG (2005). Interaction between genetic background and the mating-type locus in *Cryptococcus neoformans* virulence potential.. Genetics.

[59] Aldenderfer MS, Blashfield RK (1984). Cluster analysis..

[60] Constandse-Westermann TS (1972). Coefficients of biological distance..

[61] Sneath PH, Sokal RR (1973). Numerical Taxonomy..

[62] Sabouraud R (1896). Recherche des milieux de culture propres a la différenciation des espèces trichophytiques a grosse spore.. Les trichophyties humaines.

[63] Gardes M, Bruns TD (1993). ITS primers with enhanced specificity for basidiomycetes – application to the identification of mycorrhizae and rusts.. Molecular Ecology.

[64] White TJ, Bruns T, Lee S, Taylor JW, Innis MA, Gelfand DH, Sninsky JJ, White TJ (1990). Amplification and direct sequencing of fungal ribosomal RNA genes for phylogenetics.. PCR protocols: A guide to methods and applications.

[65] Rehner SA, Samuels GJ (1994). Taxonomy and phylogeny of *Gliocladium* analysed from nuclear large subunit ribosomal DNA sequences.. Mycological Research.

[66] Vilgalys R, Hester M (1990). Rapid genetic identification and mapping enzymatically amplified ribosomal DNA from several *Cryptococcus* species.. Journal of Bacteriology.

[67] Zoller S, Scheidegger C, Sperisen C (1999). PCR primers for the amplification of mitochondrial small subunit ribosomal DNA of lichen-forming Ascomycetes.. Lichenologist.

[68] Don R, Cox P, Wainwright B, Baker B, Mattick J (1991). ‘Touchdown’ PCR to circumvent spurious priming during gene amplification.. Nucleic Acids Research.

[69] Glenn TC, Schable NA, Zimmer EA, Roalson EH (2005). Isolating microsatelline DNA loci.. Molecular evolution: Producing the biochemical data, part B..

[70] Swofford DL (2002). Phylogenetic Analysis Using Parsimony (*and Other Methods). Version 4.

[71] Corpet F (1988). Multiple sequence alignment with hierarchical clustering.. Nucleic Acids Research.

[72] Thompson JD, Higgins DG, Gibson TJ (1994). CLUSTAL W: improving the sensitivity of progressive multiple sequence alignment through sequence weighting, positions-specific gap penalties and weight matrix choice.. Nucleic Acids Research.

[73] Kirk PM, Cannon PF, Minter DW, Stalpers JA (2008). Dictionary of the fungi..

[74] Eddy S (1998). Profile hidden Markov models.. Bioinformatics.

[75] Keller A, Schleicher T, Schultz J, Müller T, Dandekar T (2009). 5.8S-28S rRNA interaction and HMM-based ITS2 annotation.. Gene.

[76] Koetschan C, Förster F, Keller A, Schleicher T, Ruderisch B (2010). The ITS2 Database III - sequences and structures for phylogeny.. Nucleic Acids Research.

[77] Wolf M, Friedrich J, Dandekar T, Müller T (2005). CBCAnalyzer: inferring phylogenies based on compensatory base changes in RNA secondary structures.. In Silico Biology.

[78] Thompson JD, Gibson TJ, Plewniak F, Jeanmougin F, Higgins DG (1997). The CLUSTAL_X windows interface: flexible strategies for multiple sequence alignment aided by quality analysis tools.. Nucleic Acids Research.

[79] Maddison WP, Maddison DR (2009). http://mesquiteproject.org.

[80] Benson G (1999). Tandem repeats finder: a program to analyze DNA sequences.. Nucleic Acids Research.

[81] Rozen S, Skaletsky HJ, S K, Misener S (2000). Primer3 on the WWW for general users and for biologist programmers.. Bioinformatics Methods and Protocols: Methods in Molecular Biology.

[82] Geiduschek EP, Gerskovits TT (1961). Nonaqueous solutions of DNA. Reversible and irreversible denaturation in methanol.. Archives of Biochemistry and Biophysics.

