# Supplementary figures and images for: Structure, Function, and Phylogeny of the Mating Locus in the Rhizopus oryzae Complex
Source: PLoS One. 2010 Dec 9;5(12):e15273. doi: 10.1371/journal.pone.0015273 (PMC3000332; doi:10.1371/journal.pone.0015273)

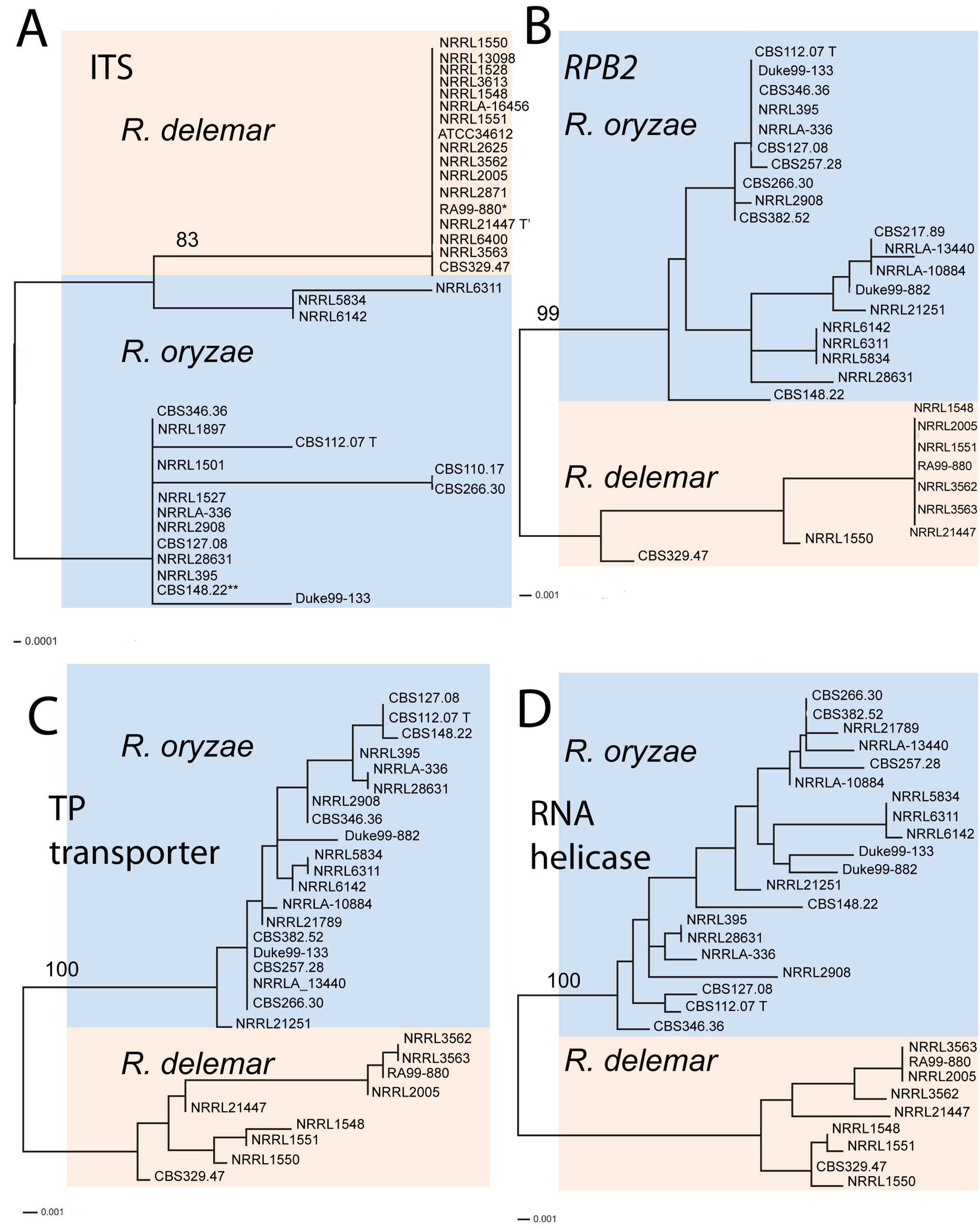

Supplement: Figure S1 — Maximum Likelihood phylogeny for rDNA ITS1-5.8S-ITS2 (A), RPB2 (B), TPT (C) and RNA helicase (D) genes. Analysis included a total of 566 (rDNA ITS1-5.8S-ITS2), 757 (RPB2), 978 (TPT) and 764 (RNA helicase) nucleotide characters. ML bootstrap proportions higher than 70 are shown above the nodes. Group * includes ITS sequences AB097299, AB181316-AB181330 of Rhizopus delemar; group ** includes the ITS sequences AB181303-AB181309, AB181311-AB181315, AB097334 of Rhizopus oryzae [10]. T – type culture of R. oryzae s. s., T' indicates a strain with an rDNA ITS1-5.8S-ITS2 sequence that is identical to the type culture of R. delemar (CBS120.12). (TIF) [file pone.0015273.s001.tif]

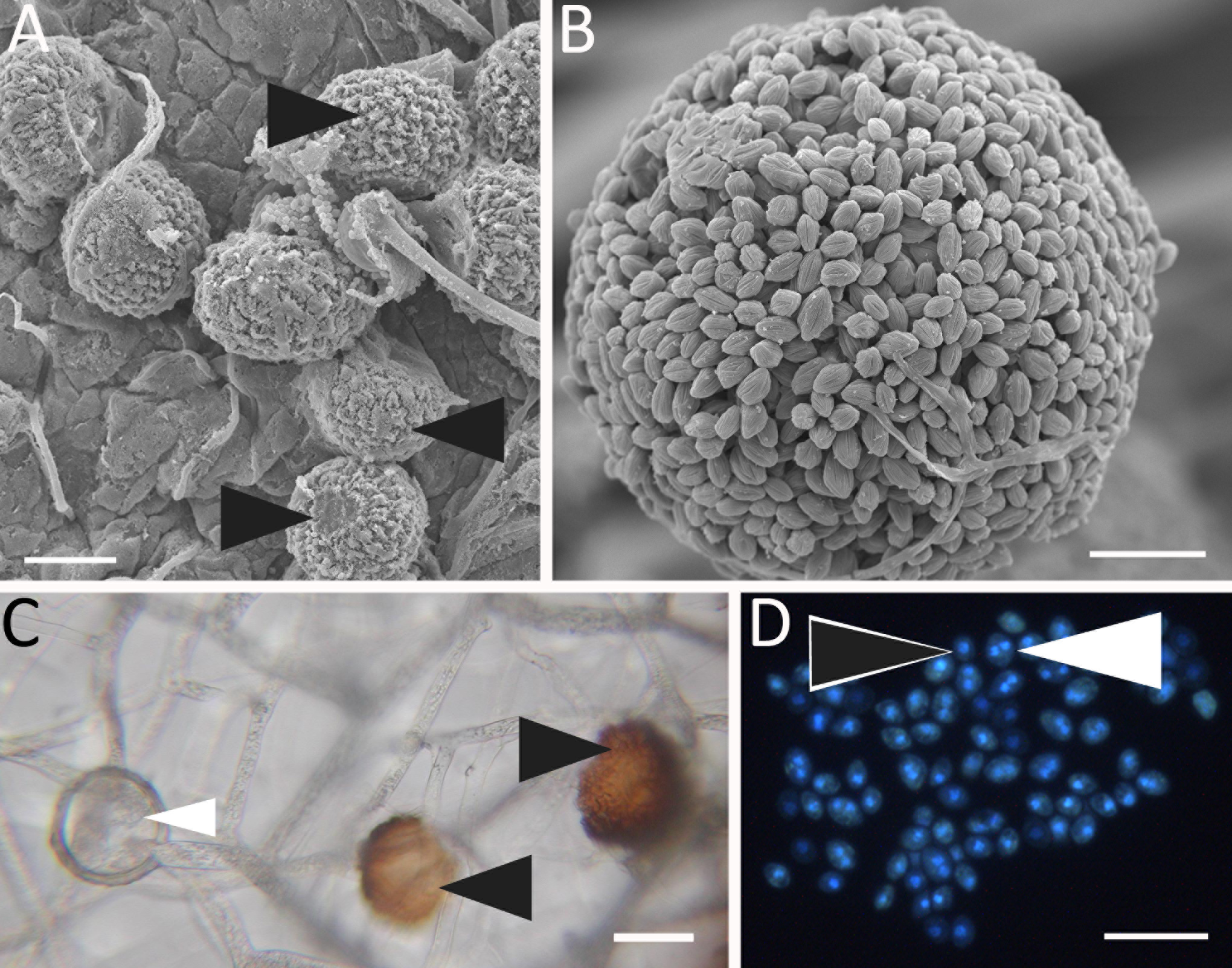

Supplement: Figure S2 — Sexual (zygospores) and asexual (sporangiospores) spores of Rhizopus oryzae. (A) Electron micrograph of a cross between R. oryzae strains CBS346.36 × CBS110.17 showing zygospores (black arrow heads). Scale bar = 50 µm. (B) Asexual sporangium of R. delemar strain NRRL3563 without sporangium wall. Scale bar = 20 µm. (C) Germinating of zygospore's protoplast (white arrow) into vegetative mycelium after crushing of lateral spore wall (black arrows). Scale bar = 50 µm. (D) Different size of uni- (black arrow) and binucleate (white arrow) DAPI stained sporangiospores of Rhizopus delemar RA99-880. Scale bar = 20 µm. (TIF) [file pone.0015273.s002.tif]

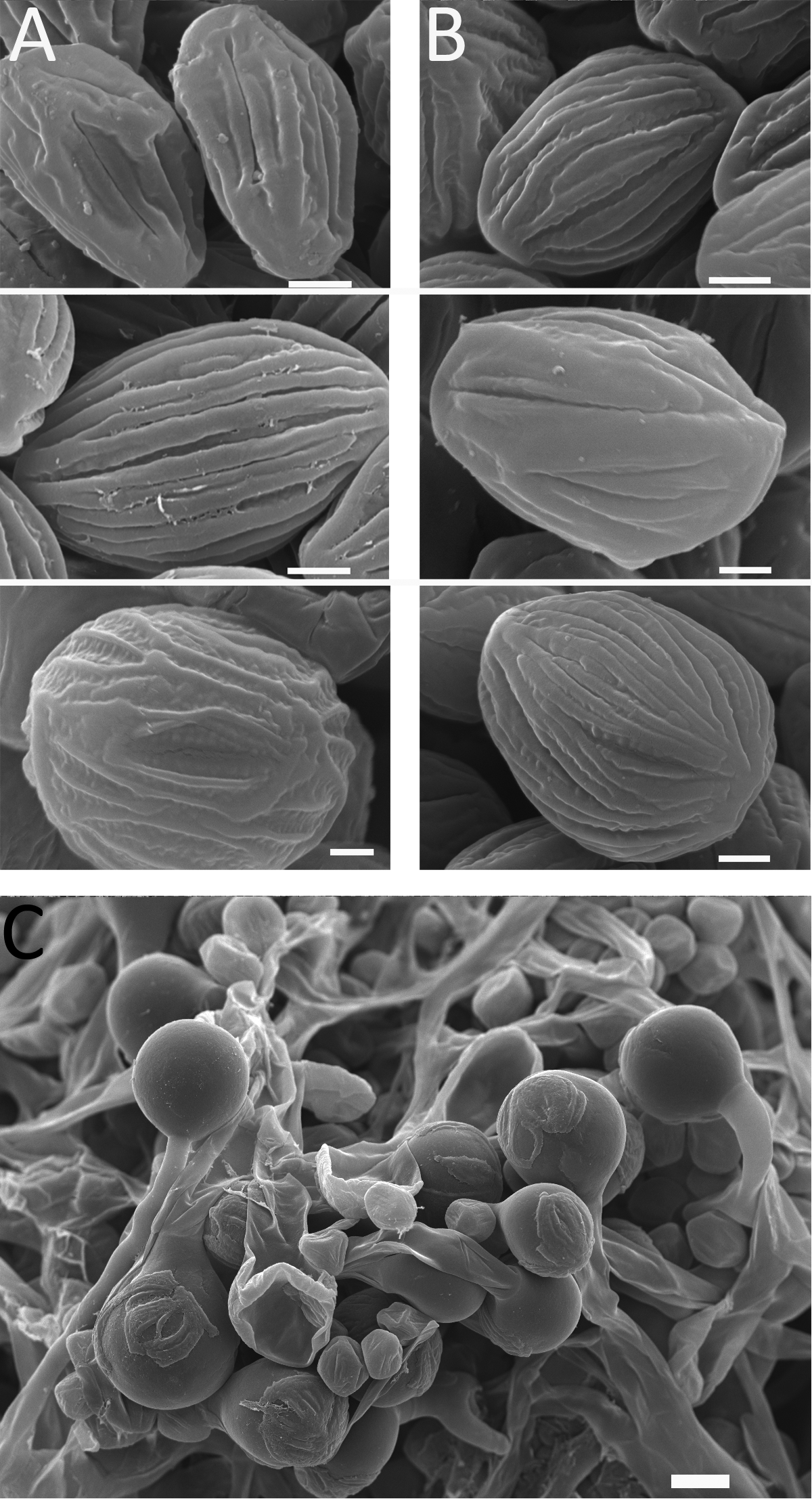

Supplement: Figure S3 — Micromorphology of sporangiospores. Panel A) Rhizopus delemar (from top to bottom): ATCC34612, RA99-880, NRRL3562. Panel B) Rhizopus oryzae s. s.(from top to bottom): NRRL2908, Duke99-133, NRRL3142. Panel C) Germination of the Rhizopus oryzae s. s. strain, CBS112.07. Scale = 1 µm for panel A and B, and 5 µm for panel C. (TIF) [file pone.0015273.s003.tif]
