# Supplementary material for: Structure, Function, and Phylogeny of the Mating Locus in the Rhizopus oryzae Complex
Source: PLoS One. 2010 Dec 9;5(12):e15273. doi: 10.1371/journal.pone.0015273 (PMC3000332; doi:10.1371/journal.pone.0015273)
Supplement: Table S1 — List of primers designed in this study. (DOC) [file pone.0015273.s004.doc]

**Table S1.** List of primers designed in this study

| *RPB2* gene | |
| --- | --- |
| Rh_RPB2_f | 5'-TGACAGACATAATGGATGACAGC-3' |
| Rh_RPB2l_r | 5'-GTAGCTGGATCTAAGCATTAC-3' |
| Rh_RPB2s_r | 5'-GCCGTTGATGACAAAGTAAC-3' |
| TP transporter | |
| RhTPtrans_for | 5'-GCCTTTGATTTGGATGAACG-3' |
| RhTPtrans_rev | 5'-CACCAGGTTGCTACAAGTCC-3' |
| Between flanking genes | |
| TPtr_RNA_1_for | 5'-TGTGCTCTAGGGTCCTGCTT-3' |
| TPtr_RNA_1_rev | 5'-AGGTTTGCTCGTGTGATTCC-3' |
| TPtr_RNA_2_for | 5'-TGTGCTCTAGGGTCCTGCTT-3' |
| TPtr_RNA_2_rev | 5'-AGAAAATACGGGTGCGTCAG-3' |
| johe20161 | 5'-ggacttgtagcaacctggtg-3' |
| johe20162 | 5'-acatcgagtttgggttttgc-3' |
| Between TP transporter and HMG box | |
| 5858-1gap_for | 5'-TGTGAAAACGATTGCACCTT-3' |
| 5858-1gap_rev | 5'-AACCAAAGAGGCACCATGAG-3' |
| 5858-3gap_for | 5'-TGATGAGCATTATTTGGTTTGG-3' |
| 5858-3gap_rev | 5'-TGTCAGCCTGATGGGAAGAT-3' |
| Rh_Sgen1plus_for | 5'-TGTCAACACTAGAGAATACATGG-3' |
| Rh_Sgen1plus_rev | 5'-CCAAGATGAAGCGTTGGATT-3' |
| Rh_Sgen2plus_for | 5'-CTCCCGAAGAAGCTGAAAGA-3' |
| Rh_Sgen2plus_rev | 5'-GCAAGTAGCGGGGAAATTCT-3' |
| Between HMG box and RNA helicase | |
| 5858-2gap_rev | 5'-GCATGAACAACAGGCGTATC-3' |
| RhizoSgene-II_for | 5'-CATCAAAAACATCAATGGCCTA-3' |
| RhizoSgene-II_rev | 5'-TTTGACTGCATTTCTCAAAGGA-3' |
| 5858-2gap_rev | 5'-GCATGAACAACAGGCGTATC-3' |
| RhizoSgene-II_for | 5'-CATCAAAAACATCAATGGCCTA-3' |
| RhizoSgene-II_rev | 5'-TTTGACTGCATTTCTCAAAGGA-3' |
| RNA helicase | |
| RhizoSGenI_for | 5'-CTGACGCACCCGTATTTTCT-3' |
| RhizoSGenI_rev | 5'-CGGGTGGACCTTGAATAATG-3' |
| RhizoSGenII_for | 5'-TGAACAACCTCTTGGTTTTGG-3' |
| RhizoSGenII_rev | 5'-CCGTGTGGGCTTTTAAGTGT-3' |
| RhizoSGenIII_for | 5'-AACCCAATTGGATGCTCTCA-3' |
| RhizoSGenIII_rev | 5'-TGAACCTGTCCATGTTTCCA-3' |
| RhizoSGenIV_for | 5'-CAAGAAACCACAGCAGCAAA-3' |
| RhizoSGenIV_rev | 5'-CTCGTCATACCAACCACACG-3' |
| RhizoSGenV_for | 5'-GCTCGATTGAAAGCACACAA-3' |
| RhizoSGenV_rev | 5'-CCATCCACACTGGATACCTG-3' |
| Rh_Hel_end_for | 5'-gcagccaagctgtcattcta-3' |
| End_helic1_rev | 5'-GGGATTCCGAGCAAGACTTC-3' |
| RhSgenInt1_rev | 5'-TGCCCTATCATTGGTCGATT-3' |
| (+) HMG box, specific and differential primers | |
| PLUS1_for | 5'-ACTAGATATGATTGACAGTAAG-3' |
| PLUS1_rev | 5'-TGGCTCCAACTGGAACAGTC-3' |
| PLUS2_for | 5'-GTAGTTCGAGTTATGTTTCCGG-3' |
| PLUS2_rev | 5'-tgcatcctgaaaactgtccc-3' |
| (-) HMG box, specific and differential primers | |
| SGeneCORE_for | 5'-CATCAAAAACATCAATGGCCTA-3' |
| SGeneCORE_rev | 5'-TAGCTCGAGAGCCACTGCTT-3' |
| Minus_for | 5'-CCGCTACTTGCAGCATGAGC-3' |
| Minus_rev | 5'-AGACGGCGGTGAAGATGGGC-3' |
| Lactate dehydrogenase genes | |
| Forward for ldhA and ldhB |  |
| johe22917 | 5'-ttggctccggtacctacctt-3' |
| Reverse for ldhA and ldhB |  |
| johe22918 | 5'-cgaatgatctccatcgcttt-3' |
| Reverse for ldhA only |  |
| johe22919 | 5'-gaaaaggaagtagcatggactttt-3' |
