# Supplementary material for: Structure, Function, and Phylogeny of the Mating Locus in the Rhizopus oryzae Complex
Source: PLoS One. 2010 Dec 9;5(12):e15273. doi: 10.1371/journal.pone.0015273 (PMC3000332; doi:10.1371/journal.pone.0015273)
Supplement: Table S2 — GenBank accession numbers of the sequences generated in this study. (DOC) [file pone.0015273.s005.doc]

**TABLE S2.** GenBank accession numbers of the sequences generated in this study

| Strains | mtSSU | LSU | ITS | *RPB2* | TPT | (+) HMG | (-) HMG | RNA helicase |
| --- | --- | --- | --- | --- | --- | --- | --- | --- |
| *R. oryzae s. s.* |  |  |  |  |  |  |  |  |
| CBS110.17* | HQ434969 | HQ434999 | HQ435049 | HQ450319 | HQ435110 | - | HQ435187 | HQ435148 |
| CBS112.07* | HQ434973 | HQ435003 | HQ435055 | HQ450325 | HQ435113 | - | HQ435191 | HQ435150 |
| CBS127.08* | HQ434989 | HQ434998 | HQ435048 | HQ450318 | HQ435112 | - | HQ435186 | - |
| CBS148.22 | - | HQ435002 | HQ435054 | HQ450324 | HQ435114 | - | HQ435190 | HQ435149 |
| CBS257.28 | - | - | HQ435067 | HQ450322 | HQ435125 | - | - | HQ435147 |
| CBS264.28 | HQ434983 | HQ435001 | HQ435053 | HQ450323 | HQ435125 | - | HQ435189 | - |
| CBS266.30 | - | HQ435041 | HQ435050 | HQ450320 | HQ435121 | - | HQ435188 | HQ435145 |
| CBS346.36* | HQ434965 | HQ434997 | HQ435047 | HQ450317 | HQ435104 | HQ435213 | - | HQ435151 |
| CBS382.52 | HQ434974 | HQ435000 | HQ435066 | HQ450321 | HQ435124 | - | - | HQ435146 |
| Duke166.02 | HQ434993 | - | HQ435071 | HQ450331 | - | HQ435217 | - | - |
| Duke99-133 | - | HQ435044 | HQ435052 | HQ450329 | HQ435123 | HQ435215 | - | HQ435152 |
| Duke99-892 | HQ434994 | HQ435045 | HQ435075 | HQ450330 | HQ435117 | HQ435216 | - | HQ435153 |
| NRRL395 | HQ434992 | HQ435009 | HQ435059 | HQ450333 | HQ435108 | HQ435226 | - | HQ435160 |
| NRRL1501 | HQ434984 | HQ435010 | HQ435060 | HQ450334 | HQ435118 | HQ435221 | - | HQ435161 |
| NRRL1510 | - | HQ435043 | HQ435061 | - | - | HQ435222 | - | - |
| NRRL1527 | HQ434967 | HQ435011 | HQ435062 | HQ450335 | HQ435109 | HQ435223 | - | HQ435162 |
| NRRL1891 | - | HQ435018 | HQ435094 | HQ450339 | - | HQ435227 | - | - |
| NRRL1897 | HQ434975 | HQ435019 | HQ435063 | HQ450337 | HQ435107 | - | HQ435193 | HQ435163 |
| NRRL2908 | - | HQ435021 | HQ435064 | HQ450338 | HQ435106 | HQ435224 | - | HQ435167 |
| NRRL3142 | - | HQ435022 | HQ435065 | - | - | - | HQ435194 | - |
| NRRL5833 | - | HQ435026 | HQ435079 | HQ450340 | - | HQ435228 | - | - |
| NRRL5834 | HQ434985 | HQ435027 | HQ435076 | HQ450341 | HQ435114 | - | HQ435195 | HQ435164 |
| NRRL6142 | HQ434988 | HQ435028 | HQ435077 | HQ450342 | HQ435120 | - | HQ435196 | HQ435165 |
| NRRL6257 | - | HQ435040 | HQ435057 | - | - | - | - | - |
| NRRL6311 | HQ434996 | HQ435031 | HQ435078 | HQ450343 | HQ435116 | - | HQ435197 | HQ435166 |
| NRRL6431 | - | HQ435033 | HQ435072 | - | - | - | HQ435198 | - |
| NRRL10206 | HQ434979 | HQ435007 | HQ435070 | HQ450328 | - | HQ435220 | - | - |
| NRRL21251 | HQ434991 | HQ435042 | HQ435068 | HQ450326 | HQ435127 | HQ435218 | - | HQ435157 |
| NRRL21789 | HQ434971 | HQ435005 | HQ435069 | HQ450327 | HQ435122 | HQ435219 | - | HQ435154 |
| NRRL28631 | - | HQ435038 | HQ435051 | HQ450336 | HQ435111 | HQ435225 | - | HQ435159 |
| NRRLA-336 | HQ434986 | HQ435008 | HQ435058 | HQ450332 | HQ435105 | - | HQ435192 | HQ435158 |
| NRRLA-10884 | HQ434977 | HQ435034 | HQ435073 | HQ450345 | HQ435126 | - | HQ435199 | HQ435156 |
| NRRLA-13142 | - | HQ435035 | HQ435056 | - | - | - | HQ435200 | - |
| NRRLA-13440 | HQ434990 | HQ435036 | HQ435074 | HQ450344 | HQ435119 | - | HQ435201 | HQ435155 |
| *R. delemar* |  |  |  |  |  |  |  |  |
| ATCC34612 | HQ434972 | HQ435039 | HQ435103 | HQ450364 | HQ435144 | HQ435238 | - | HQ435185 |
| CBS329.47 | - | - | HQ435080 | HQ450346 | HQ435128 | - | HQ435202 | HQ435168 |
| NRRL1528 | HQ434978 | HQ435012 | HQ435081 | HQ450347 | HQ435131 | - | HQ435203 | HQ435175 |
| NRRL1547 | - | HQ435013 | HQ435082 | HQ450348 | - | HQ435229 | - | - |
| NRRL1548 | - | HQ435014 | HQ435083 | HQ450349 | HQ435129 | - | HQ435205 | HQ435176 |
| NRRL1549 | - | HQ435015 | HQ435084 | - | - | HQ435230 | - | HQ435177 |
| NRRL1550 | HQ434968 | HQ435016 | HQ435085 | HQ450350 | HQ435132 | - | HQ435209 | HQ435178 |
| NRRL1551* | HQ434970 | - | HQ435086 | HQ450351 | HQ435130 | - | HQ435210 | HQ435179 |
| NRRL1552 | - | HQ435017 | HQ435087 | HQ450352 | - | - | HQ435211 | - |
| NRRL2005 | HQ434982 | - | HQ435093 | HQ450353 | HQ435137 | HQ435231 | - | HQ435169 |
| NRRL2625 | HQ434976 | - | HQ435088 | HQ450354 | HQ435141 | HQ435232 | - | HQ435170 |
| NRRL2871 | HQ434966 | HQ435020 | HQ435089 | HQ450355 | HQ435142 | HQ435233 | - | HQ435171 |
| NRRL3562 | HQ434995 | HQ435023 | HQ435090 | HQ450356 | HQ435139 | HQ435234 | - | HQ435172 |
| NRRL3563 | HQ434987 | HQ435024 | HQ435091 | HQ450357 | HQ435138 | HQ435235 | - | HQ435173 |
| NRRL3613 | HQ434964 | HQ435025 | HQ435092 | HQ450358 | HQ435143 | HQ435236 | - | HQ435174 |
| NRRL6201 | - | HQ435029 | HQ435098 | - | - | - | HQ435206 | - |
| NRRL6202 | - | HQ435030 | HQ435099 | - | - | - | HQ435207 | - |
| NRRL6400 | - | HQ435032 | HQ435100 | HQ450360 | HQ435140 | HQ435237 | - | HQ435180 |
| NRRL13098 | - | - | HQ435101 | HQ450361 | HQ435135 | - | HQ435208 | HQ435181 |
| NRRL21447 | HQ434963 | HQ435004 | HQ435096 | HQ450363 | HQ435134 | - | HQ435204 | HQ435183 |
| NRRLA-16456 | HQ434981 | HQ435037 | HQ435102 | HQ450362 | HQ435133 | - | HQ435212 | HQ435182 |
| RA99-880* | HQ434980 | HQ435006 | HQ435097 | HQ450365 | HQ435136 | HQ435239 | - | HQ435184 |
| *R. microsporus* |  |  |  |  |  |  |  |  |
| Duke133.05 | - | HQ435046 | HQ450314 | - | - | - | - | - |
