# Supplementary material for: Structure, Function, and Phylogeny of the Mating Locus in the Rhizopus oryzae Complex
Source: PLoS One. 2010 Dec 9;5(12):e15273. doi: 10.1371/journal.pone.0015273 (PMC3000332; doi:10.1371/journal.pone.0015273)
Supplement: Table S3 — Isolates of the Rhizopus oryzae complex and related species used in this study; isolates of Rhizopus oryzae and Rhizopus delemar are named according to their placement in phylogenetic trees (See Results). (DOC) [file pone.0015273.s006.doc]

**Table S3**. Isolates of the *Rhizopus oryzae* complex and related species used in this study; isolates of *Rhizopus oryzae* and *Rhizopus delemar* are named according to their placement in phylogenetic trees (See Results).

| **Collection #** | **Zygospore production** | **Mating type** | **Origin and notes** |
| --- | --- | --- | --- |
| ***R. oryzae sensu stricto*** |  |  |  |
| CBS110.17^3^ | yes | minus ^1, 2^ | as *R. maydis*, human pathogen |
| CBS112.07^3, 4, 5^ | yes | minus ^1, 2^ | human pathogen |
| CBS127.08^3^ | yes | minus ^1, 2^ | human pathogen |
| CBS148.22 | yes | minus ^1, 2^ | as *R. tonkinensis*, lactic acid |
| CBS257.28^5^ | yes | minus ^1, 2^ | as *R. formosaensis*, fermented food |
| CBS264.28 | yes | minus ^1, 2^ | as *R. pseudochinensis*, Chinese yeasts |
| CBS266.30 | yes | minus ^1, 2^ | as *R. fusiformis*, on *Brassica naphobrassica* root |
| CBS346.36^3, 5^ | yes | plus ^1, 2^ | non pathogenic |
| CBS382.52 | yes | minus ^1, 2^ | produces steroids |
| Duke166.02 | no | plus ^2^ | human pathogen |
| Duke99-133 | no | n. d. | human pathogen |
| Duke99-892 | no | plus ^2^ | human pathogen |
| NRRL395 | no | plus ^2^ | tempeh |
| NRRL1501 | no | plus ^2^ |  |
| NRRL1510 | no | plus ^2^ |  |
| NRRL1527 | no | plus ^2^ | air |
| NRRL1891 | no | plus ^2^ | Hildebrandt |
| NRRL1897 | yes | minus ^1, 2^ | grain |
| NRRL2908 | yes | plus ^1, 2^ | Chinese yeasts |
| NRRL3142 | no | minus ^2^ | Chinese yeasts |
| NRRL5833 | no | plus ^2^ | produces 6-azauridine |
| NRRL5834 | no | minus ^2^ | barley |
| NRRL6142 | no | minus ^2^ | parsnip, produces steroids |
| NRRL6257 | no | n. d. | tempeh |
| NRRL6311 | no | minus ^2^ | soft red wheat |
| NRRL6431 | no | minus ^2^ | cotton seeds |
| NRRL10206 | no | plus ^2^ | human pathogen |
| NRRL21251 | no | plus ^2^ | human pathogen |
| NRRL21789 | yes | plus ^2^ | human pathogen |
| NRRL28631 | no | plus ^2^ | human pathogen |
| NRRLA-336 | yes | minus ^1, 2^ | wine cake |
| NRRLA-10884 | no | minus ^1^ | human pathogen |
| NRRLA-13142 | no | minus ^2^ | human pathogen |
| NRRLA-13440 | yes | minus ^1, 2^ | human pathogen |
| ***R. delemar*** |  |  |  |
| ATCC34612 | no | plus ^2^ | on *Brassica pekinensis* root |
| CBS329.47 | yes | minus ^1, 2^ | tempeh, produces pectinase |
| NRRL1528 | no | minus **^2^** |  |
| NRRL1547 | no | plus **^2^** | cotton roots |
| NRRL1548 | yes | minus **^1, 2^** |  |
| NRRL1549 | no | plus **^2^** |  |
| NRRL1550 | yes | minus **^1, 2^** |  |
| NRRL1551^3^ | no | minus **^2^** |  |
| NRRL1552 | no | minus **^2^** |  |
| NRRL2005 | no | plus **^2^** | produces fumaric acid |
| NRRL2625 | no | plus **^2^** | produces starch, amylolysis |
| NRRL2871 | no | plus **^2^** | as *R. jawanicus* |
| NRRL3562 | no | plus **^2^** | tempeh |
| NRRL3563 | no | plus **^2^** | tempeh |
| NRRL3613 | no | plus **^2^** | tempeh |
| NRRL6201 | no | minus **^2^** | tempeh |
| NRRL6202 | no | minus **^2^** | tempeh |
| NRRL6400 | no | plus **^2^** | wine cake, produces fumaric acid |
| NRRL13098 | no | minus **^2^** | tapioca (tapai-ubi) |
| NRRL21447 | no | minus **^2^** | human pathogen |
| NRRLA-16456 | no | minus **^2^** | human pathogen |
| RA99-880^3^ | no | plus **^2^** | human pathogen |
| ***R. microsporus*** |  |  |  |
| Duke133.05 | no | n. d. |  |
| ***Phycomyces blakesleeanus*** | |  |  |
| NRRL1555^3^ | yes | minus **^1, 2^** |  |
| UBC21^3^ | yes | plus **^1, 2^** |  |
| ***Mucor cincinelloides*** |  |  |  |
| R7B^3^ | yes | minus **^1, 2^** |  |
| NRRL3161^3^ | yes | plus **^1, 2^** |  |

^1^determined in mating assays

^2^determined by sequencing the *sex* locus

^3^Strains for which the sex locus and flanking genes have been sequenced

^4^Type culture

^5^Tester strains used in mating assays

n. d. – not determined
